# Polymorphism of Butyl Ester of Oleanolic Acid—The Dominance of Dispersive Interactions over Electrostatic

**DOI:** 10.3390/ijms24076572

**Published:** 2023-03-31

**Authors:** Dominik Langer, Barbara Wicher, Zbigniew Dutkiewicz, Wioletta Bendzinska-Berus, Barbara Bednarczyk-Cwynar, Ewa Tykarska

**Affiliations:** 1 Department of Chemical Technology of Drugs, Poznan University of Medical Sciences, Grunwaldzka 6, 60-780 Poznan, Poland; 2 Department of Organic Chemistry, Poznan University of Medical Sciences, Grunwaldzka 6, 60-780 Poznan, Poland

**Keywords:** triterpenes, oleanolic acid esters, monotropic polymorphs, intermolecular energies calculations, intermolecular interaction, hydrogen bonds, hydrophobic interactions, differential scanning calorimetry, crystal structures

## Abstract

Oleanolic (OA) and glycyrrhetinic acids (GE), as well as their derivatives, show a variety of pharmacological properties. Their crystal structures provide valuable information related to the assembly modes of these biologically active compounds. In the known-to-date crystals of OA esters, their 11-oxo derivatives, and GE ester crystals, triterpenes associate, forming different types of ribbons and layers whose construction is based mainly on van der Waals forces and weak C-H···O interactions. New crystal structures of 11-oxo OA methyl ester and the polymorph of OA butyl ester reveal an alternative aggregation mode. Supramolecular architectures consist of helical chains which are stabilized by hydrogen bonds of O-H···O type. It was found that two polymorphic forms of butyl OA ester (layered and helical) are related monotropically. In a structure of metastable form, O-H···O hydrogen bonds occur, while the thermodynamically preferred phase is governed mainly by van der Waals interactions. The intermolecular interaction energies calculated using CrystalExplorer, PIXEL, and Psi4 programs showed that even in motifs formed through O-H···O hydrogen bonds, the dispersive forces have a significant impact.

## 1. Introduction

The polymorphic phenomenon is well known for organic compounds [1,2,3,4]. It is of particular interest to the pharmaceutical industry, since polymorphs might differ in solubility, dissolution rate, bioavailability, stability, hardness, melting point, etc. [5,6,7]. Thus, discovering polymorphic forms of active substances cannot be overestimated, but it is not a straightforward task. Factors such as impurities, additives, solution saturation, solution mixtures, thermodynamics, and many others can influence crystallization processes and are difficult to control [2,8,9]. Numerous methods are employed for polymorph screening, e.g., recrystallization, slurrying, cooling crystallization, sublimation, lyophilization, grinding, compression, or template-inducing nucleation [10,11,12,13,14]. These days, experimental methods are also supported by computational techniques, but still, McCrone’s statement from the year 1965, “…in general, the number of forms known for a given compound is proportional to the time and money spent in research on that compound” is valid [15].

Triterpenes are a well-known class of natural compounds found in various medicinal plants, for instance, oleanolic acid in *Olea europaea* leaves [16], boswellic acid in *Boswellia sacra* resin [17], and glycyrrhizic and glycyrrhetinic acids in *Glycyrrhiza glabra* root [18]. Tens of thousands of triterpene structures have been identified [19,20]. In modern medicinal chemistry, pentacyclic triterpenes, especially glycyrrhetinic (GE) [21] and oleanolic acids (OA) [22], are used to develop new effective drugs. GE, OA, and their derivatives possess a variety of biological activities, including anti-inflammatory [23,24,25,26,27], antiviral [28,29], anticancer [30,31,32], cytostatic, or proapoptotic ones [26,32,33].

**Scheme 1:**
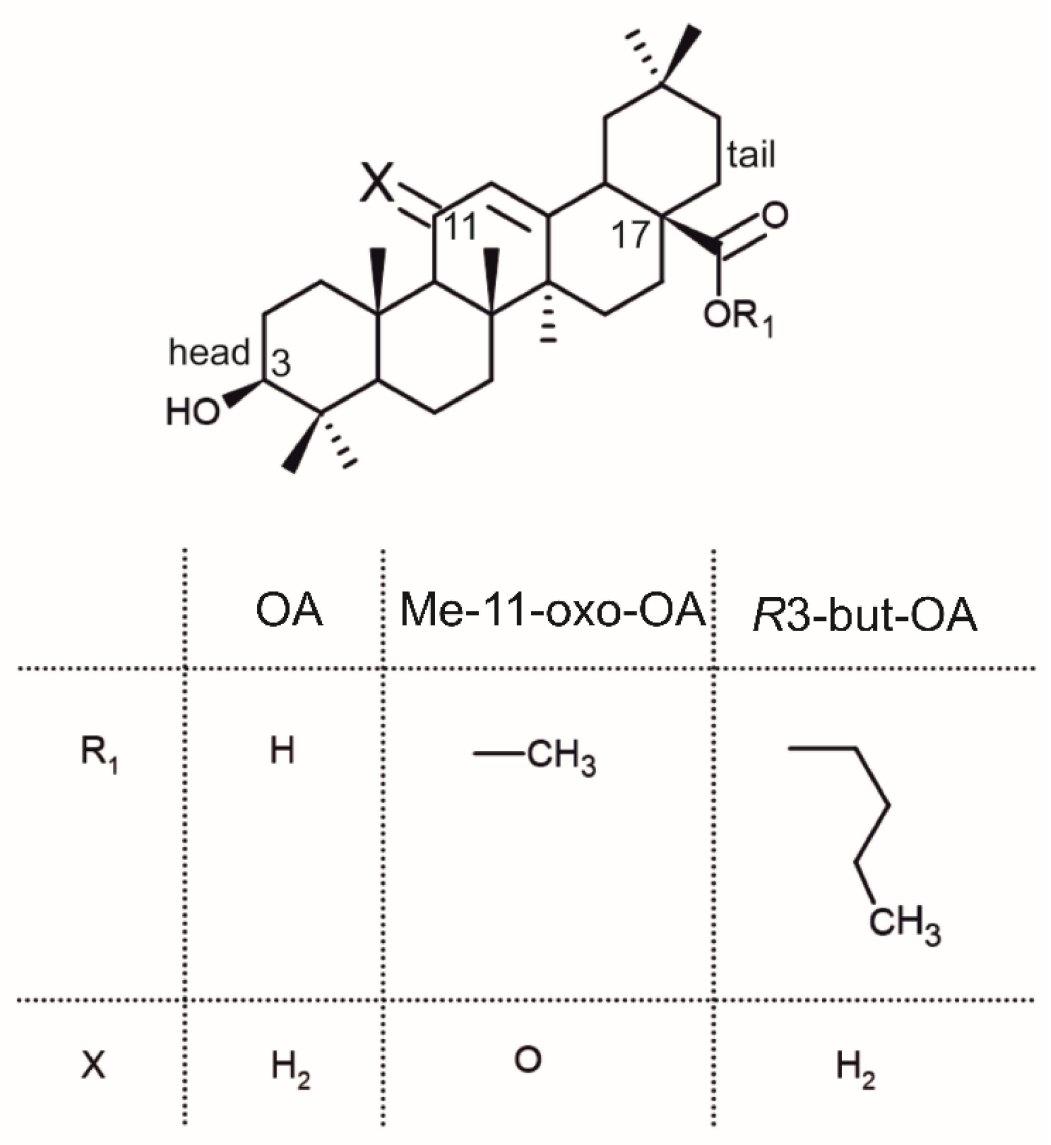
OA and its derivatives chemical structures.

The OA molecule comprises a rigid pentacyclic triterpenoid core decorated with hydroxyl and carboxylic substituents. The molecule’s elongated shape allows the head and tail to be distinguished (Scheme 1). 

**Figure 1:**
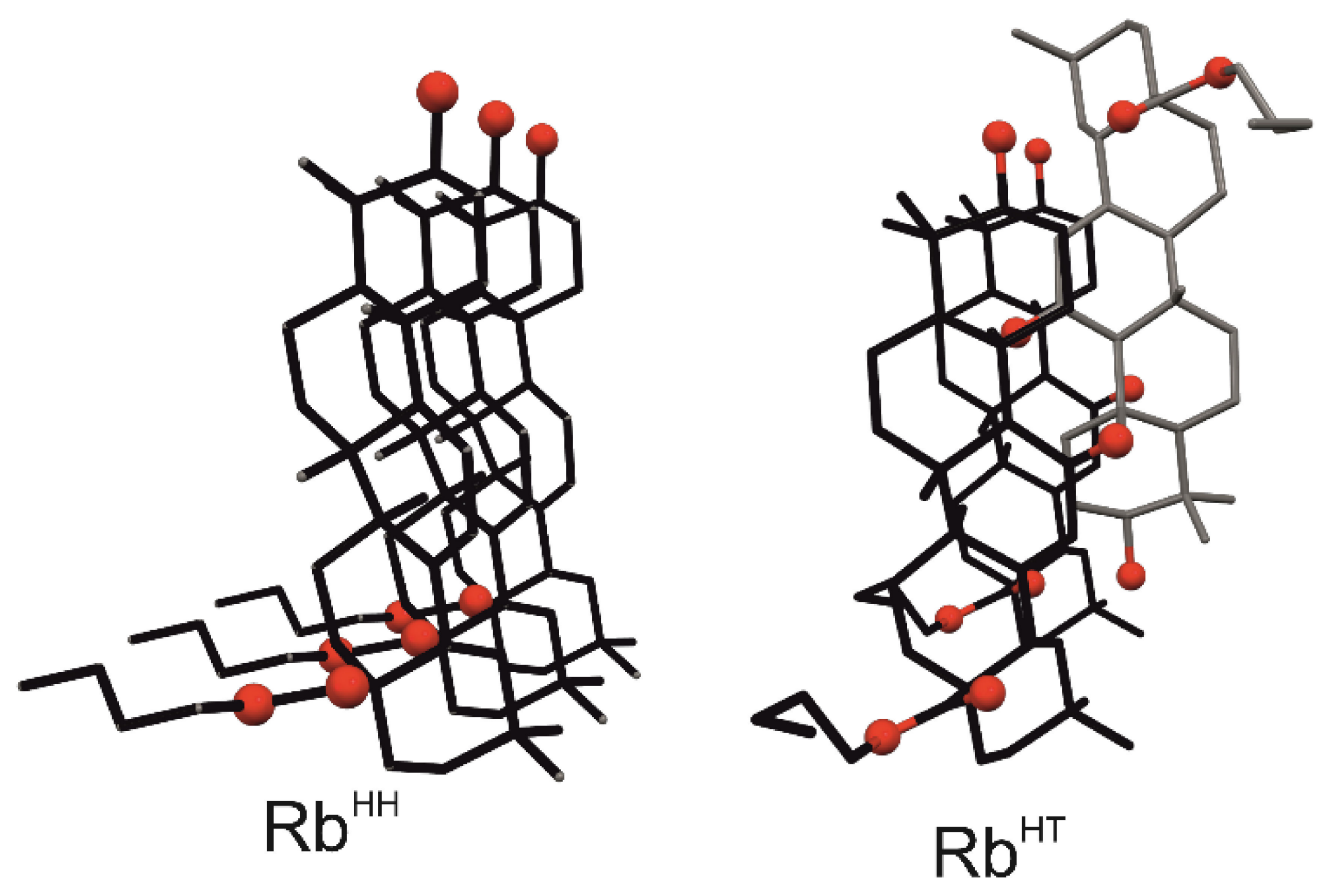
The head-to-head (Rb^HH^) and head-to-tail (Rb^HT^) ribbons. C-atoms of triterpene skeletons related by 2_1_ screw axes in the Rb^HT^ are colored in black and gray [34].

In our recent publication related to crystal structures of esters of OA and its 11-oxo analogues [34], we showed that despite structural diversity among crystals, the association of molecules can be described by only two schemes. Triterpenes aggregate (1) head-to-head, forming flat ribbons Rb^HH^ where subsequent molecules are related by translation, or (2) head-to-tail, creating zigzag ribbons Rb^HT^ with molecules related by a two-fold screw axis (Figure 1). These 1D motifs assembly into layers of two types that are characteristic of each kind of ribbon. Similar 1D and 2D motifs are also basic structural units in crystal structures of GE solvates, GE esters, and carbenoxolone [9,35,36,37,38,39].

It has been noticed that layered crystal structures of a variety of OA esters and their 11-oxo analogues are isostructural, proving that substituents at the triterpene core (both ester moiety and O11 carbonyl) do not necessarily influence crystal packing [34]. In this context, the structures of butyl esters of OA and its 11-oxo analogue are very intriguing. The latter compound crystallizes in two polymorphic forms, in which Rb^HT^ ribbons are the basic structural motifs of the layer. In contrast, the layered crystal structure of the butyl ester of OA consists of Rb^HH^. So, a few questions arise: Is it possible to obtain another polymorph for OA butyl ester? Is the formation of Rb^HT^ ribbons possible in crystals of OA butyl ester? To answer these questions, we performed a series of recrystallization experiments on OA butyl ester. Here, we present the crystal structure of its new polymorph and the calculations of intermolecular interaction energy governing crystal structures. Moreover, a comparison of this new form with the known structure of GE methyl ester solvate [35] and, published here for the first time, the structure of the solvated crystal of methyl ester of 11-oxo OA derivative are provided.

## 2. Results and Discussion

**Figure 2:**
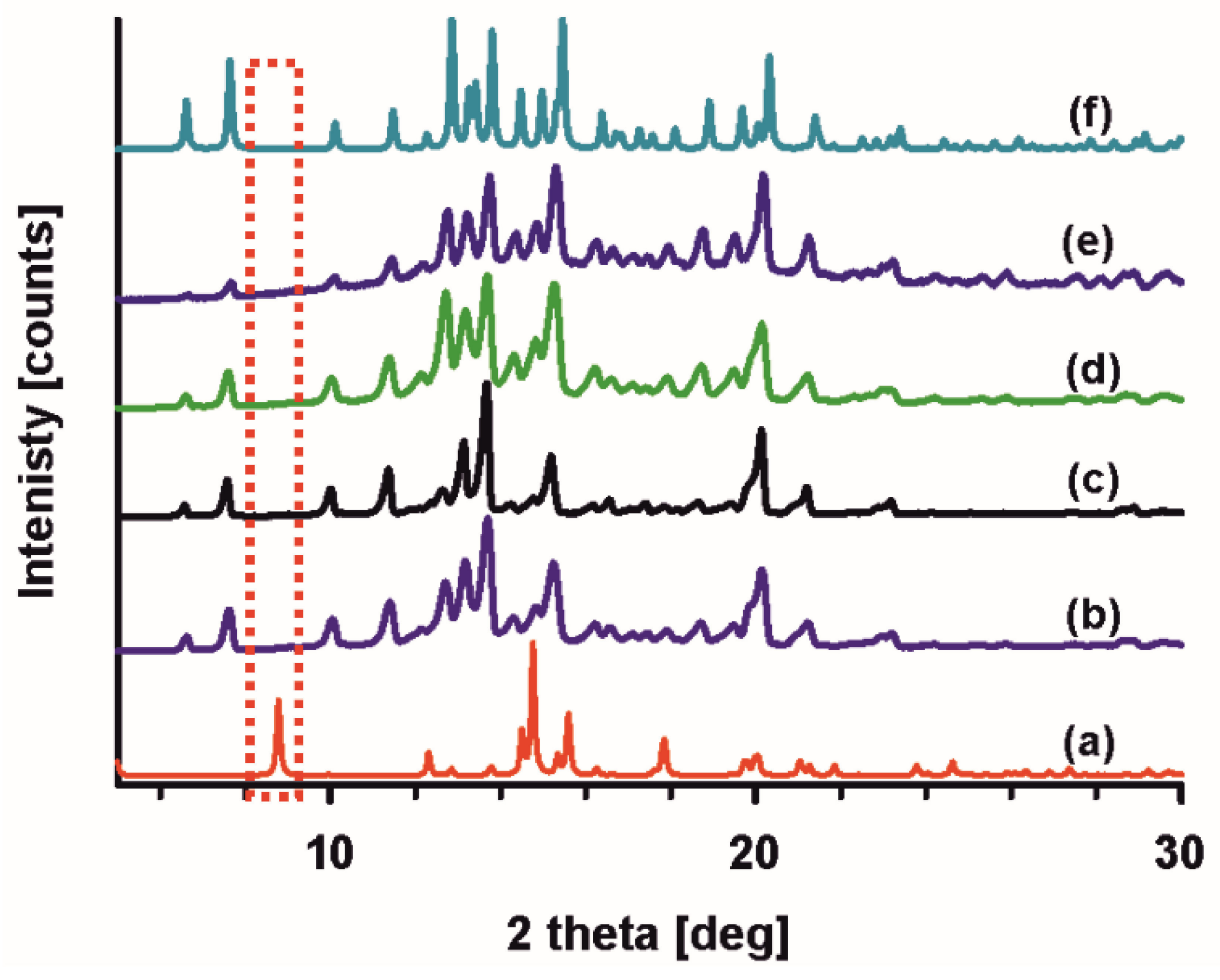
Powder diffraction patterns of but-OA simulated from single crystals belonging to (**a**) *P*2_1_ and (**f**) *R*3 space groups, obtained during recrystallization from (**b**) ethyl acetate, (**c**) 2-propanol, (**d**) ethanol, and (**e**) dimethylsulfoxide. The intensity characteristic for the *P*2_1_ form is marked with a red, dotted rectangle.

The polymorphism of 11-oxo OA butyl ester [34] prompted us to search for different crystalline forms of butyl OA ester. We started screening for polymorphs by dissolving the crystalline *P*2_1_ form of ester (***P*2_1_-but-OA**) in various solvents. Powder X-ray diffraction data were collected for recrystallized samples. As shown in Figure 2, all experimental powder patterns are identical but differ from the simulated diffractogram of a *P*2_1_ single-crystal structure [34]. These results indicate that a new phase was obtained.

With these results in hand, we set up many crystallization experiments to obtain a single crystal of a new phase. It turned out to be a difficult task, since only powder samples were received. Eventually, a crystal grown from dimethylformamide was sufficient for the X-ray diffraction experiment. The obtained crystal, further denoted as ***R*3-but-OA**, belongs to the high-symmetry space group *R*3. The simulated powder diffraction pattern based on this structure proved that it is the form received during screening experiments (Figure 2).

### 2.1. Crystal Structure of R3-but-OA versus P2_1_-but-OA

**Figure 3:**
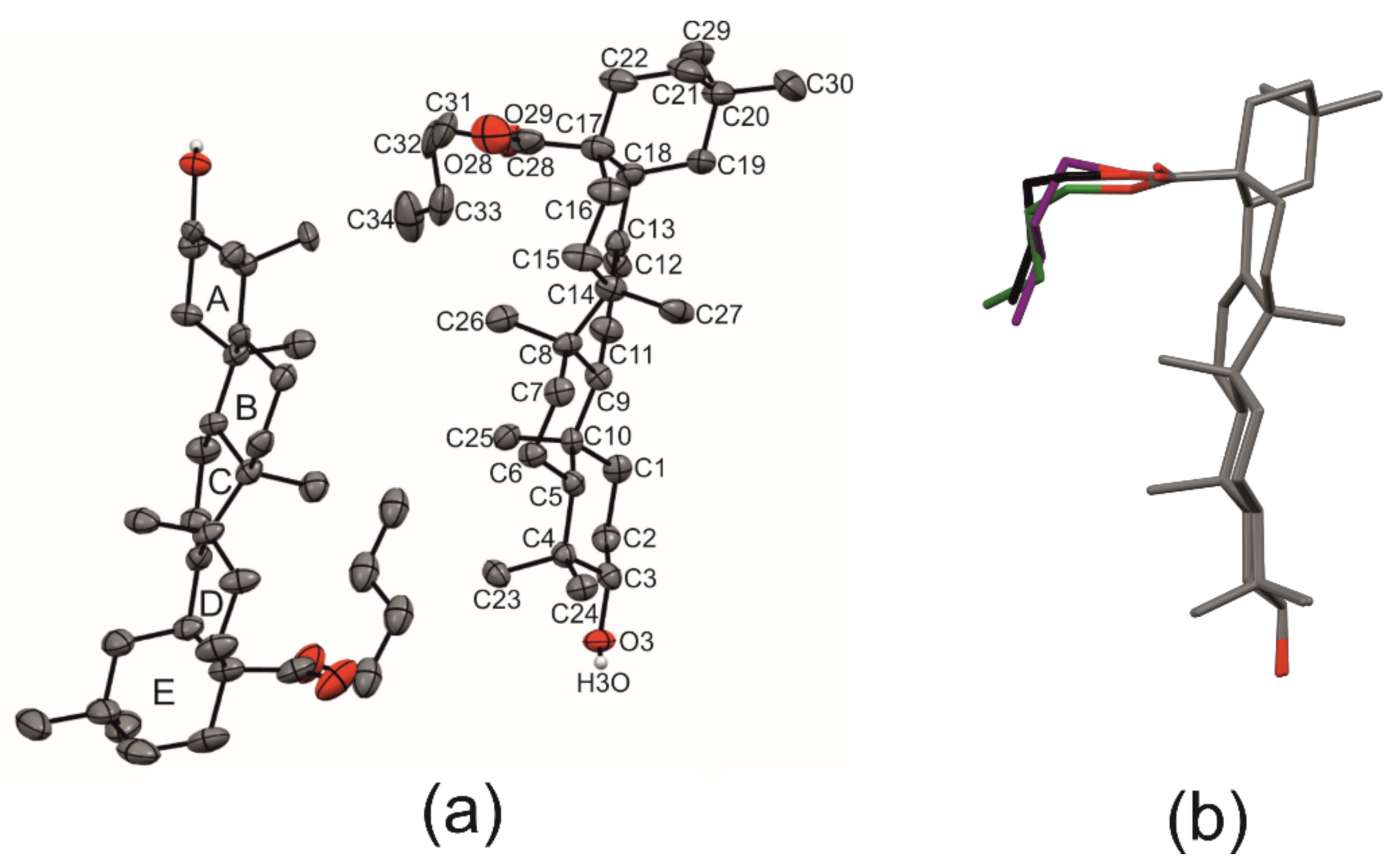
(**a**) ORTEP representation of an asymmetric unit of ***R*3-but-OA**: For molecule A, atoms are labeled; the numeric scheme for molecule B is the same. For molecule B, only the major position of the disordered butyl substituent is shown. Except for these from the O-H group, the H atoms are omitted for clarity. Displacement ellipsoids are at a 50% probability level. (**b**) Superposition of two symmetry-independent ester molecules in ***R*3-but-OA**. The butyl substituent of molecule A is colored green, violet major (66.2%), and black minor (33.8%) positions of molecule B.

***R*3-but-OA** has two symmetry-independent molecules of OA butyl ester, A and B, in the asymmetric unit (Figure 3a, Table S1). The structures of both molecules are nearly identical. Minor differences are observed for the butyl substituent, which in B is disordered (Figure 3b). The torsion angles C18-C17-C28-O28_carbonyl,_ which indicate the rotation of ester substituent around a single C-C bond connecting it to the triterpene skeleton, are 164.7° and 153.2° in A and B molecules, respectively. The corresponding torsion angle in the *P*2_1_ phase (***P*2_1_-but-OA**) is −21.5°, indicating that ester groups are rotated by approximately 180° in these two polymorphs [34]. Despite these differences, the energy of molecules calculated using the B3LYP/6-31G (d,p) level basis set in *Gaussian09, Revision. A.02* software [40] is nearly identical: −975,729.5 kcal/mol (molecule A), −975,727.8 kcal/mol (molecule B, major occupancy of butyl substituent), −975,723.1 kcal/mol (molecule B, minor occupancy) in ***R*3-but-OA**, and −975,733.9 kcal/mol (the molecule in ***P*2_1_-but-OA**).

**Figure 4:**
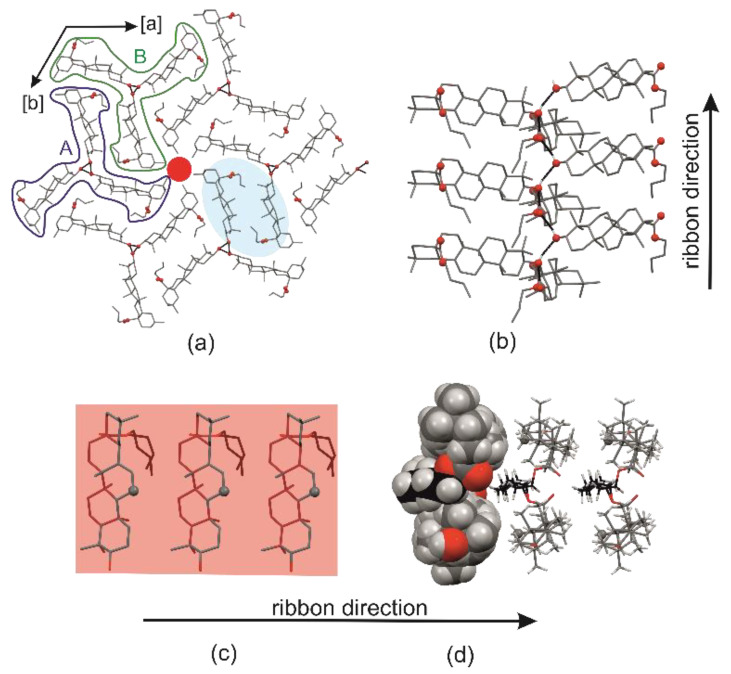
***R*3-but-OA** (**a**) three-dimensional structure, (**b**) helix, (**c**) Rb^HH^ ribbon of molecule B, and (**d**) A/B ribbon dimer, shown along triterpene long axis. In (**a**), the symmetry-independent propeller-shaped H-bonded helices are marked with blue and green lines; voids between E rings are marked with red circles; and the ribbon dimer is shaded with a blue ellipse. The O-H···O hydrogen bonds are shown as black lines in (**a**,**b**). In (**c**), the C11 atoms in C rings are shown as balls; the ribbons’ plane is shown in light red. In (**a**–**c**), H atoms are omitted for clarity. The butyl substituents are colored black in (**c**,**d**).

In ***R*3-but-OA,** triterpenes aggregate, forming two symmetry-independent propeller-like helices consisting of either A or B molecules related with three-fold screw axes 3_2_ and 3_1_, respectively (Figure 4a). Helices arise as a result of hydrogen bonding between triterpene hydroxyl O3 groups (Table 1). These OH groups are in the core of the helix, while triterpene backbones protrude outside, forming the blades of the propeller. The long axes of molecules are practically perpendicular to the axis of the helix (Figure 4a,b). Each blade consists of head-to-head organized molecules, related by translation along the helix axis (unit cell parameter c of ca. 7.3 Å). This flat 1D motif was already distinguished in other OA derivatives and, as earlier, is named head-to-head ribbon—Rb^HH^ (Figure 4c). Since symmetry-independent helices consist of A or B molecules, the ribbons of each helix are referred to as ribbons A and B, respectively.

The 3D structure of ***R*3-but-OA** emerges via van der Waals interactions between ribbons of adjacent propeller-like helices (Figure 4a). It leads to the formation of A/B ribbon dimers with ribbons organized head-to-tail (Figure 4a,d). The ribbons within the dimer are not shifted in the ribbon’s long-axis direction (Figure 4d) but are slightly displaced in the triterpene’s long-axis direction (Figure 4a). The butyl groups point towards the interior of the dimer. Each dimer is surrounded by six other ribbons from four different dimers (Figure 4a). The small voids are formed between rings E (Figure 4a), but thermogravimetry (Figure S1) confirmed that no solvent is present in crystals.

The motif of a flat Rb^HH^ ribbon is also observed in the *P*2_1_ crystal structure of the butyl OA ester. However, the mode of association of these 1D units is different, and the supramolecular architectures of the two polymorphs show no 2D and 3D similarities. In ***P*2_1_-but-OA**, ribbons assemble head-to-tail into layers with the hydroxyl O3-H group buried in the layer’s hydrophobic interior. This sole strong hydrogen bond donor does not participate in any hydrogen bonding; only weak C-H···O hydrogen bonds and van der Waals interactions between molecules occur [34].

Although the structures of the two polymorphs of butyl OA ester differ, it is possible to compare the construction of ribbons using parameter R, which was introduced in our earlier publications concerning crystal structures of GE derivatives [38,39]. R is a dihedral angle between the plane of the ribbon (forty-four C atoms of fused rings of two translation-related triterpene skeletons in the ribbon) and the plane defined by the practically coplanar five atoms of ring C (C9, C11-C14). Virtually, it represents the C ring position relative to the ribbon plane and indicates, to some extent, the rotation of the molecule about its long axis. Additionally, the signs (−) and (+) of the parameter R mean that the C11 atom of the C ring is on the opposite or the same side of the ribbon, respectively, as the ester group.

The value of R for the ribbon in the *P*2_1_ polymorph is (−)49.2°, while in the *R*3 phase, it is (−)2.8° for ribbon A and (−)5.0° for B (Table S2). It means that in each polymorph, ester molecules stack differently within the ribbon, but symmetry-independent ribbons in *R*3 are almost identical. Low R values in the ***R*3-but-OA** indicate that ring C is nearly parallel to the ribbon plane (Figure 4c). The minus sign denotes that the C11 atom and ester substituent lie on opposite sides of the ribbon plane. Changes in the rotation of the molecules within the ribbon influence the interactions between triterpene molecules. Ribbon B in ***R*3-but-OA** is based only on van der Waals interactions. A weak C-H···O hydrogen bond between the carbonyl O28 atom and methyl group of butyl substituent supports ribbon A. In ***P*2_1_-but-OA**, the ribbon is also strengthened with weak C-H···O interactions, but between the O28 atom and CH_2_ group of triterpene core (Table S3).

### 2.2. Differential Scanning Calorimetry (DSC) and Intermolecular Interaction Energy of OA Butyl Ester Polymorphs

**Figure 5:**
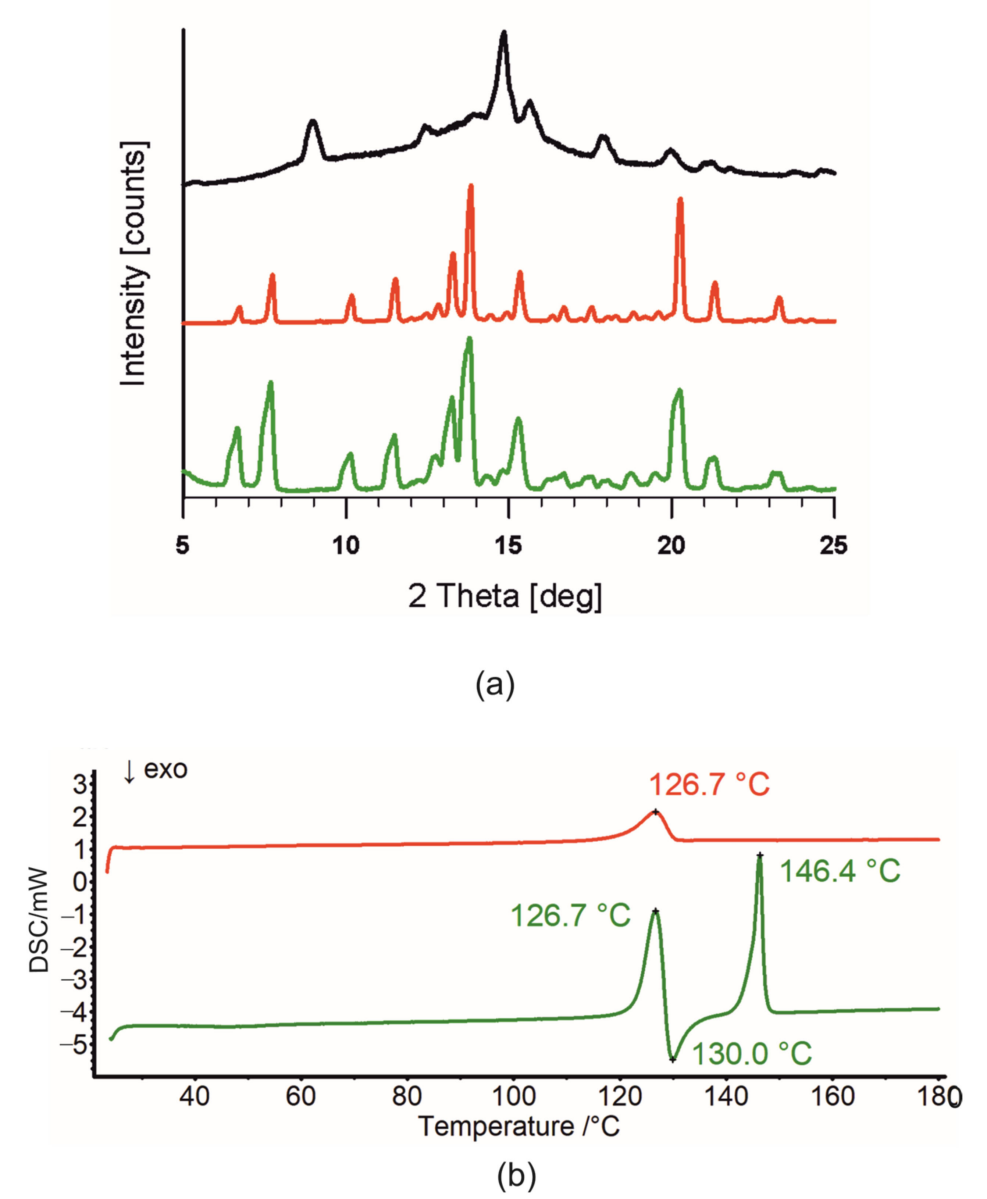
Green and red PXRD diffractograms (**a**) of ***R*3-but-OA** samples used for DSC measurements shown in (**b**). The black pattern in (**a**) was registered after heating of “green” sample to 130 °C. The red PXRD pattern corresponds to the red thermogram and green to green.

When a polymorphic phenomenon occurs, there is always the question of the thermodynamic relationship between the two forms. The earlier published *P*2_1_ polymorph thermogram shows one melting endotherm at 146.3 °C [34]. For the *R*3 phase, the DSC measurement was preceded by the powder diffraction experiment, which corroborates the phase purity of the ***R*3-but-OA** (Figure 5a, red diffractogram). The DSC thermogram (Figure 5b, red curve) showed only one endothermic event, the melting point of ***R*3-but-OA** at 126.7 °C (T_onset_ = 121.9 °C). The heat of fusion is 24.8 kJ/mol for ***P*2_1_-but-OA** and 23.4 kJ/mol for ***R*3-but-OA**. According to the “heat of fusion rule”, these two polymorphs are monotropically related [41]. Surprisingly, the second DSC thermogram (Figure 5b, green curve) registered for a different crystalline sample of pure *R*3 phase (Figure 5a, green diffractogram) varies and displays three thermal events. Taking into account the temperature maxima of these effects, they are the melting endotherm of ***R*3-but-OA** (126.7 °C), followed by crystallization of the melt (130.0 °C), and the melting of the *P*2_1_ polymorph (146.4 °C). A possible reason for the distinct thermal properties of these two samples is that the traces of the *P*2_1_ phase below the PXRD sensitivity are present in the second sample, and the nucleus promotes its crystallization from the melt.

To corroborate this assumption, a sample for which three thermal events are seen on the thermogram was heated at 130 °C for 30 min. The PXRD pattern (Figure 5, black diffractogram) registered afterwards confirms that ***P*2_1_-but-OA** and amorphous phases are arising.

Slurry experiments also confirmed the thermodynamic stability of the ***P*2_1_-but-OA** polymorph. The starting ***R*3-but-OA** phase transformed into ***P*2_1_-but-OA** within two weeks (Figure S2).

**Figure 6:**
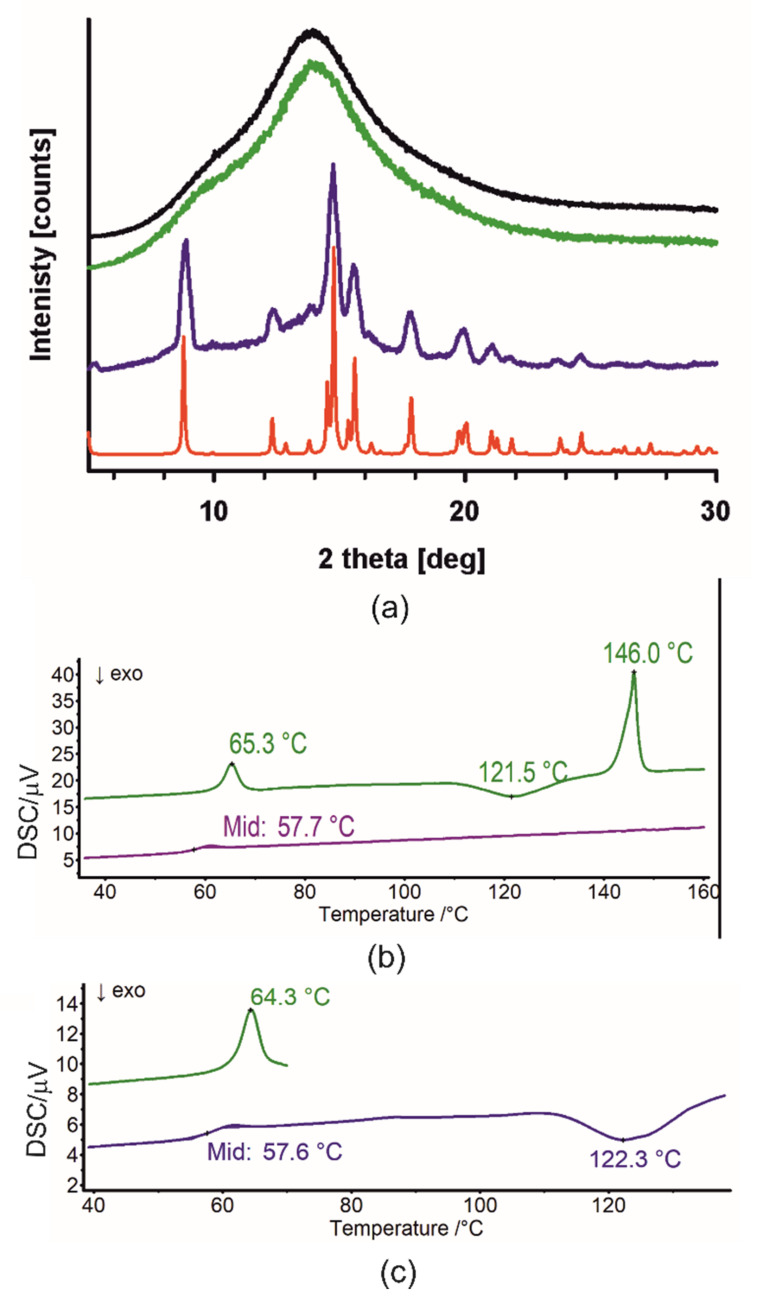
PXRD patterns (**a**) of the but-OA ester: starting amorphous sample (black), after heating to 70 °C (green) and 138 °C (purple), and simulated based on the ***P*2_1_-but-OA** crystal structure (red). DSC thermograms (**b**,**c**) of the amorphous form of the but-OA ester: green 1st heating and purple 2nd heating.

Thermal behavior for the amorphous form obtained directly after synthesis was also checked. The lack of crystallinity was confirmed with the PXRD diffractogram (Figure 6a, black). Figure 6b shows the DSC thermogram of this amorphous sample in two heating cycles. Three thermal events were observed during the first heating, endothermic with a peak at 65.3 °C, followed by exothermic (peak at 121.5 °C) and second endothermic, with a maximum at 146.0 °C (Figure 6b green), while only glass transition was observed for the second heating cycle (Figure 6b purple). The temperatures of the last two effects of the first heating are similar to those surveyed for the crystalline form (Figure 5b, green); so, they could be related to the crystallization and melting of ***P*2_1_-but-OA**. The origin of the first endotherm was unclear. Thus, another set of DSC supported by PXRD experiments was performed, i.e., after heating on the DSC apparatus sample was cooled, and the PXRD pattern was registered.

First, the amorphous sample was heated to 70 °C, and as expected, one endotherm with a maximum of 64.3 °C was observed (Figure 6c green). The powder diffractogram shows that the sample remains amorphous (Figure 6a, green). This sample was next heated to 138 °C. At the thermogram, the glass transition at 57.6 °C and the very broad exotherm with the maximum at 122.3 °C were observed (Figure 6c, dark blue). The PXRD pattern shows the emergence of the ***P*2_1_-but-OA** polymorph (compare Figure 6a, purple and red). This experiment proved that the first endotherm is the relaxation of the amorphous material that completely masks the glass transition characterized by slight inflection, i.e., small Cp changes. As initially suspected, the following two thermal events (Figure 6b green) are the crystallization and melting of the ***P*2_1_-but-OA** polymorph. Additionally, the lack of crystallization during the second heating cycle indicates that the nucleus of the ***P*2_1_-but-OA** polymorph is needed for this process.

These conclusions are also supported by the movie recorded while heating the amorphous sample at the heating apparatus. First, the amorphous state’s vitrification is observed. Then, the crystallization occurs, followed by the melting (Video S1).

The DSC results show that a polymorph **R3-but-OA**, where O-H···O hydrogen bonds and dispersion interactions are observed, is the metastable phase, while in the thermodynamically preferred form ***P*2_1_-but-OA**, the latter interactions mainly occur. These observations suggest that the forces responsible for stabilizing the crystal structures of OA derivatives are mainly dispersive.

The intermolecular energy calculations were performed with CrystalExplorer [42] and PIXEL [43] programs and the Psi4 package [44] to support experimental data. Details concerning the theoretical analysis are given in Appendix A. However, what is worth mentioning is that results obtained with CrystalExplorer and PIXEL are in very good agreement with those received with the ωB97M-V/def2-TZVPD level of theory [45,46].

In the text below, energy values calculated with CrystalExplorer are discussed for motifs governed mainly by dispersive forces. For O-H···O helices, the energies from ωB97M-V/def2-TZVPD models are given.

**Figure 7:**
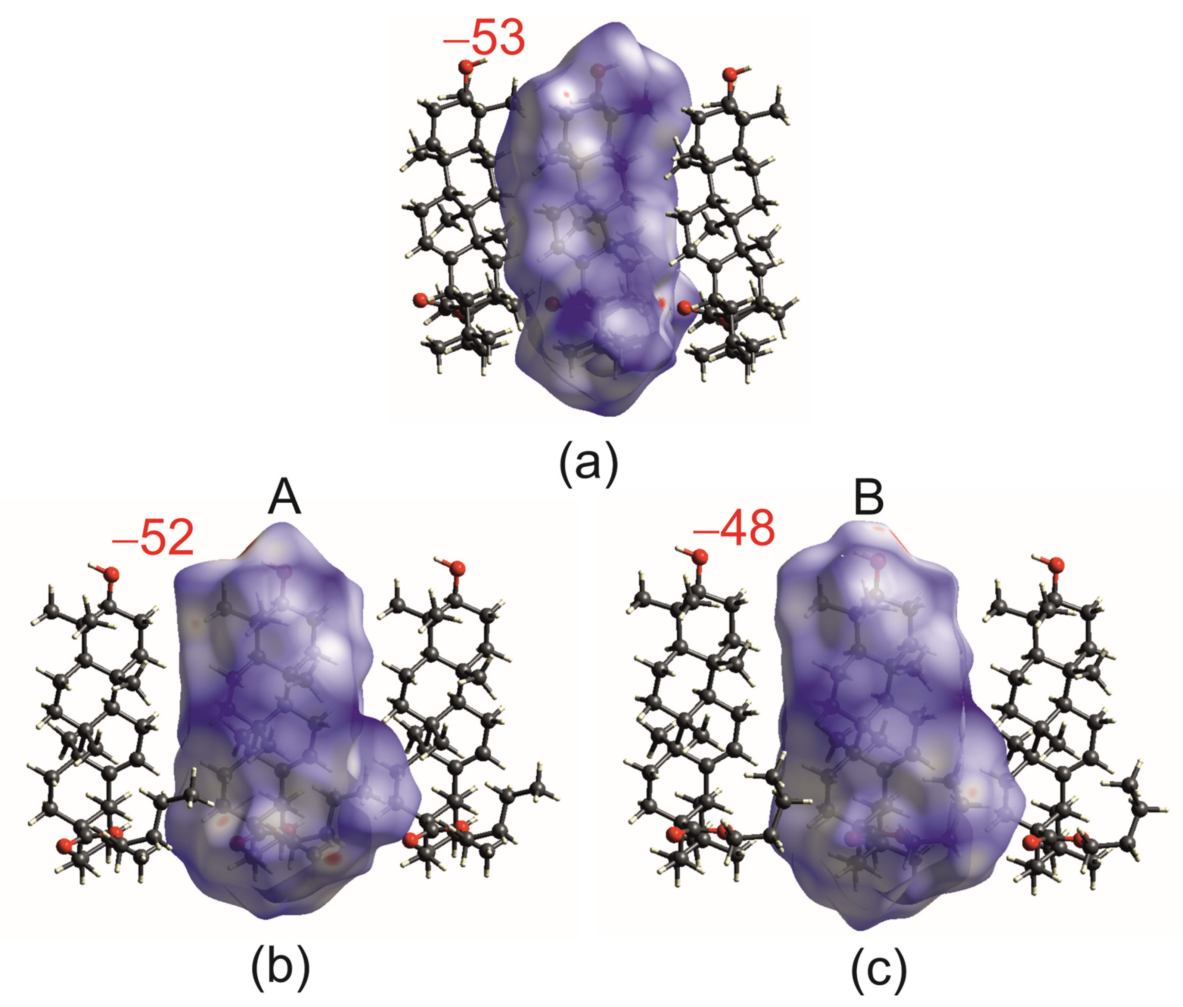
Intermolecular energies (kJ/mol) between molecules in R^HH^ ribbons in (**a**) ***P*2_1_-but-OA** and (**b**) ***R*3-but-OA** molecule A, (**c**) ***R*****3-but-OA** molecule B. For one molecule, Hirshfeld surfaces mapped with d_norm_ are shown. The energy values color match the color in Table S4.

Intermolecular energy calculations revealed that in both polymorphs, interactions between molecules in the ribbon are the most energetically favored, with energy values around −51 kJ/mol (Figure 7). In all three distinguished R^HH^, the dispersion component is dominant. However, the electrostatic part and overall stabilization energy are slightly elevated for those supported with C-H···O hydrogen bonds (Figure 7a,b and Figure S3, Table S4).

**Figure 8:**
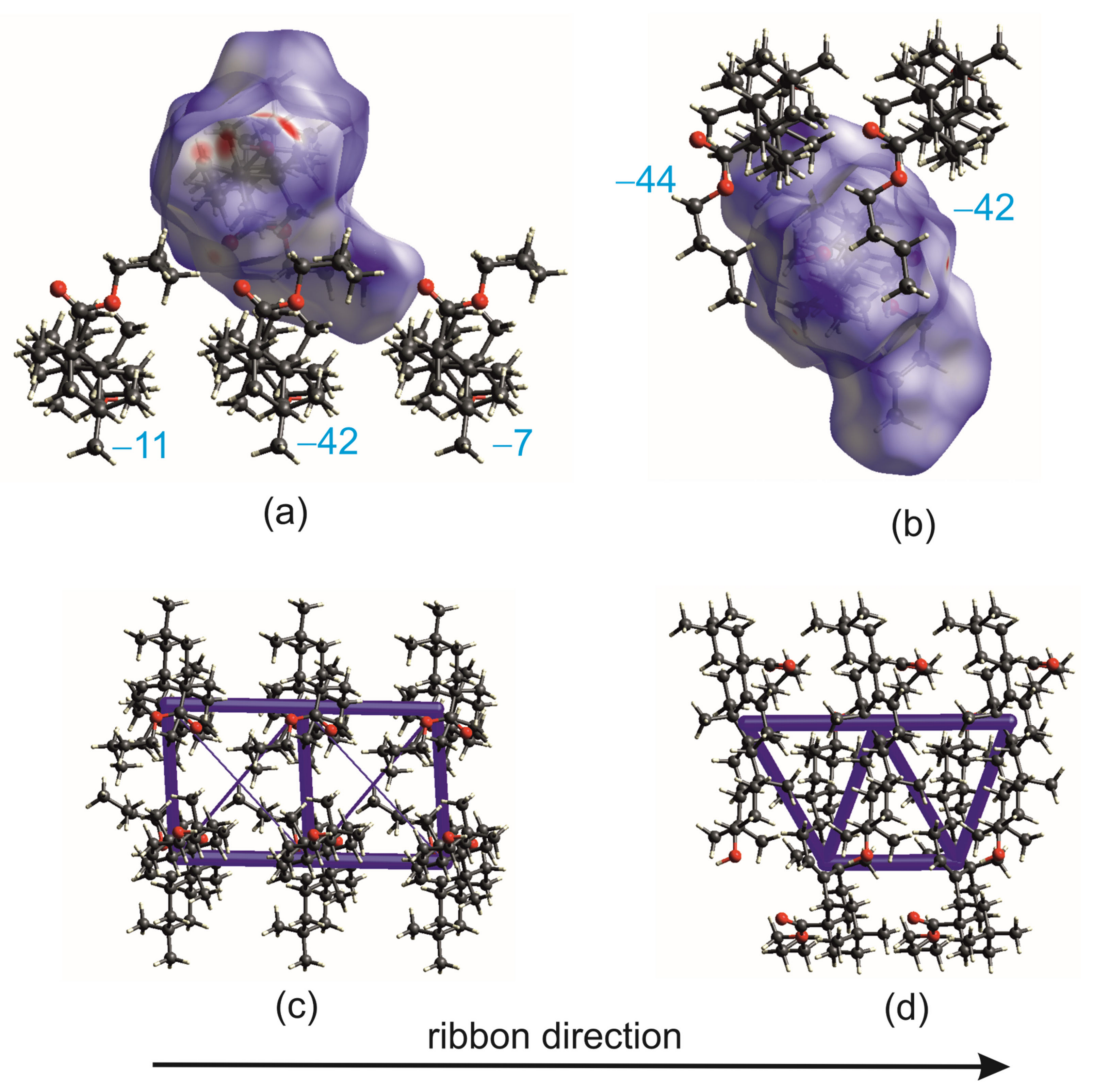
Intermolecular energies (kJ/mol) between molecules within the ribbon dimer and total energy frameworks in (**a**,**c**) ***R*3-but-OA** and (**b**,**d**) ***P*2_1_-but-OA**: in (**a**,**b**) for one molecule, Hirshfeld surfaces mapped with d_norm_ are shown, and the energy values color match the color in Table S4. Interaction energies in (**c**,**d**) are shown as cylindrical tubes with diameters proportional to the magnitude of the energies.

The intermolecular interaction energies between molecules from neighboring ribbons in a dimer depend on the relative displacement of triterpenes. In the ***R*3-but-OA** polymorph, the energy between the nearest molecules is −42 kJ/mol, but the energy between subsequent molecules drops significantly and is only −11 kJ/mol and −7 kJ/mol (Figure 8a,c and Figure S3, Table S4). In the ***P*2_1_-but-OA**, the layer consists of head-to-tail-organized R^HH^ ribbons; thus, the structural unit, such as the ribbon dimer, might be distinguished as a part of the layer (Figure S4). However, this motif’s architecture differs from the dimer observed in ***R*3-but-OA**. Interaction energies for adjacent molecules are −44 kJ/mol and −42 kJ/mol, proving that the arrangement of the molecules into layers is energetically well-stabilized (Figure 8b,d).

**Figure 9:**
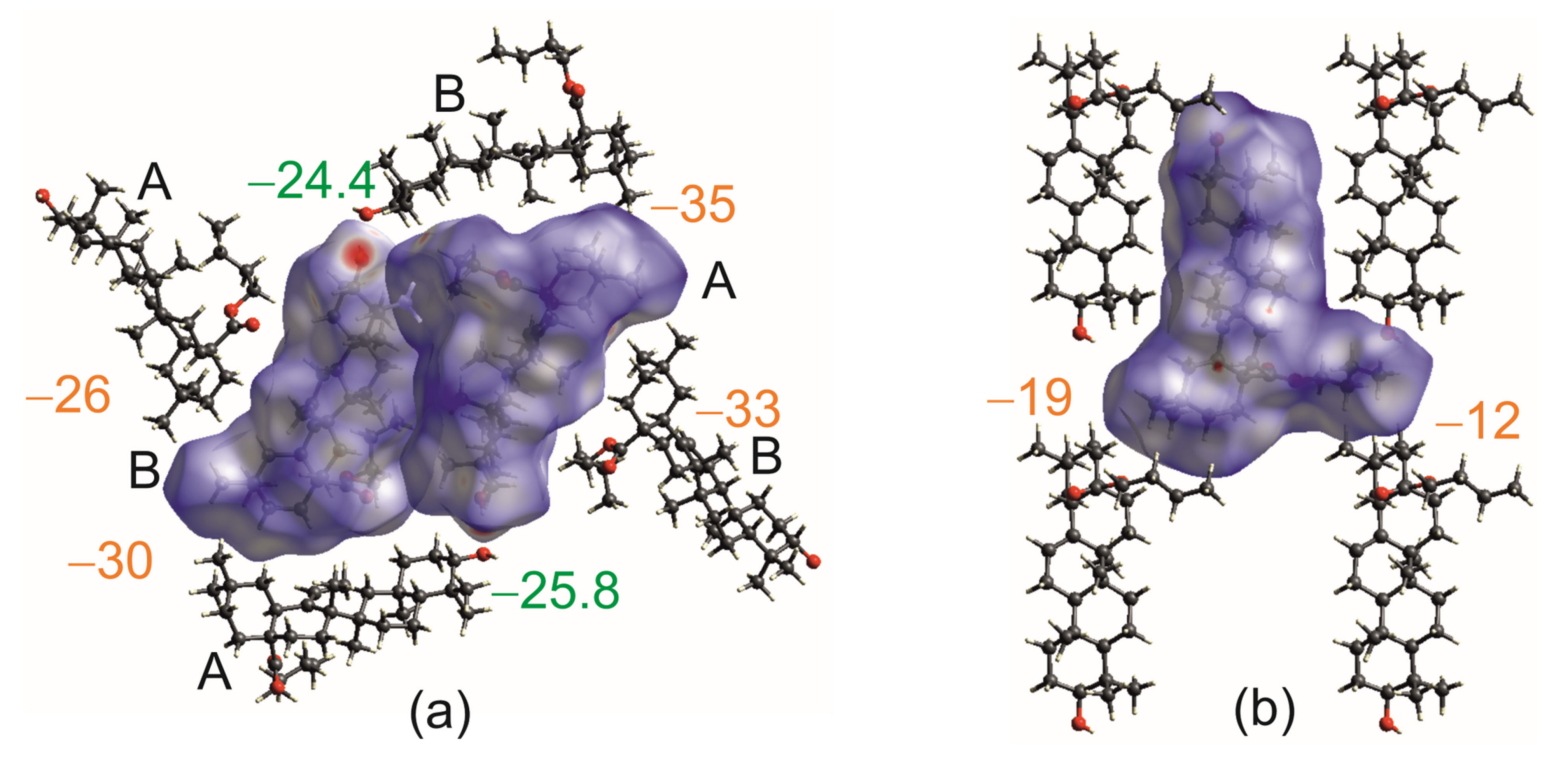
The energy of the (**a**) O-H···O hydrogen bonds (green) and side interaction (orange) of molecules from dimer to molecules from surrounding ribbons in ***R*3-but-OA** and (**b**) interlayer interaction in ***P*2_1_-but-OA**: The energy values color match the color in Table S4. For selected molecules, Hirshfeld surfaces mapped with d_norm_ are shown. For O-H···O hydrogen bonds, the given values were calculated with ωB97M-V/def2-TZVPD calculations.

The O-H···O hydrogen bonds in ***R*3-but-OA** that bind molecules into helical chains have energy of −25.8 (mol. A) and −24.4 kJ/mol (mol. B), which is almost 30 kJ/mol less than the interaction energy between molecules in R^HH^ (Figure S3). As can be seen from energy frameworks (Figure S5), the electrostatic and dispersive components for these interactions are comparable (Figure 9a, Table S4). Interestingly, similar values of total energy, but with predominant dispersive parts, are for interactions of the molecules from the dimer with neighboring molecules not bound by hydrogen bonds (Figure 9a).

The mainly dispersive interlayer interaction energies in ***P*2_1_-but-OA** have values of −19 kJ/mol and −12 kJ/mol (Figure 9b).

The intermolecular interactions’ energy calculations showed that in both polymorphic phases, the energetically most favored interactions are between molecules in the ribbon. The most significant differences are seen in the interaction energies between molecules from neighboring ribbons in a dimer. In the ***P*2_1_-but-OA**, triterpene molecules in this motif are firmly bound, contrary to those in ***R*3-but-OA** (Figure 8). It could be concluded that ribbon dimers are better stabilized in ***P*2_1_-but-OA** form than in ***R*3-but-OA**. These differences might be the factor that determined the thermodynamic preference of the ***P*2_1_-but-OA** polymorph over ***R*3-but-OA**.

The other might be crystal packing. The molecules in a thermodynamically stable form are more tightly packed. There are no free spaces, as calculated with Mercury [47]. In ***R*3-but-OA**, small voids are observed. It is reflected in the packing coefficient of 68.1 and 67.2 for ***P*2_1_-but-OA** and ***R*3-but-OA**, respectively. So, it could be concluded that layers ensure more effective crystal packing than helical structures.

### 2.3. Crystal Structures with Self-Assembly Schemes Similar to R3-but-OA

The molecular assembly mode in ***R*3-but-OA** differs from that observed in other OA esters [34]. However, this kind of packing is not new, as it occurs in a solvated crystal of methyl ester of glycyrrhetinic acid, **Me-GE** [35]. During crystallization experiments on OA and 11-oxo-OA esters, we obtained solvated crystals of methyl ester of 11-oxo OA derivative, **Me-11-oxo-OA**, which also represents similar self-assembly to ***R*3-but-OA**.

#### 2.3.1. GE Methyl Ester Solvate [35]

**Figure 10:**
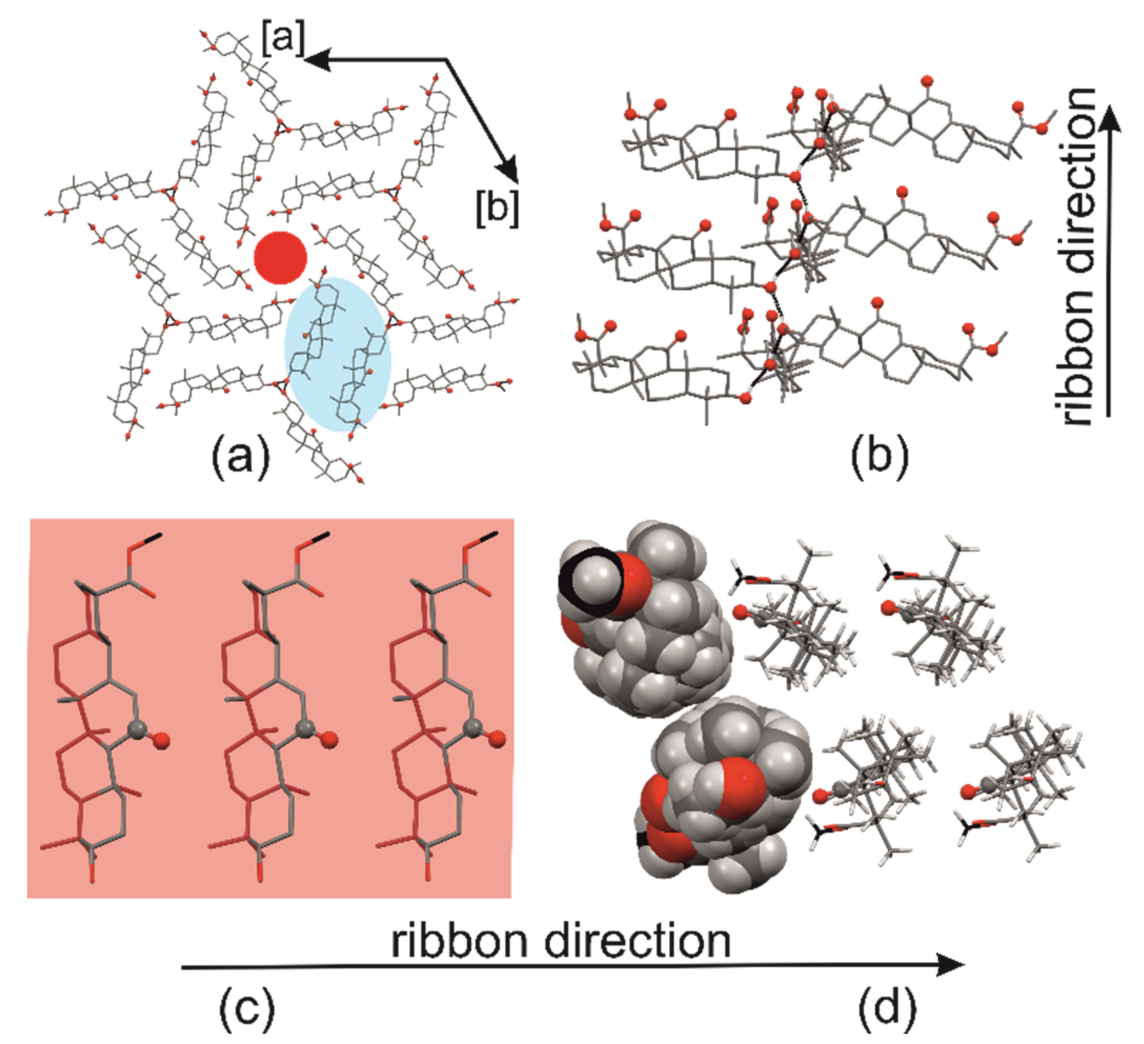
**Me-GE:** (**a**) three-dimensional structure, (**b**) helix, (**c**) Rb^HH^ ribbon, and (**d**) ribbon dimer shown along triterpene long axis: In (**a**), voids between E rings are marked with red circles; the ribbon dimer is shaded with a blue ellipse. The O-H···O hydrogen bonds are shown as black lines in (**a**,**b**). In (**c**), the C11 and O11 atoms in C rings are shown as balls; the ribbons’ plane is shown in light red. In (**a**–**c**), H atoms are omitted for clarity. The methyl substituents are colored black in (**c**,**d**). Figures were prepared based on a structure with refcode FULJOM [48].

Glycyrhetinic acid differs from OA with the position of the ester group (at C30 of the ring E) and the additional oxo group at the C11 atom (Figure S6). The crystals of **Me-GE** published by Beseda et al. [35] belong to the *P*6_5_ space group, with one molecule in the asymmetric unit. However, the supramolecular architectures of **Me-GE** and ***R*3-but-OA** can be described by similar structural motifs. Hydroxyl groups of methyl ester of GE form hydrogen-bonded propeller-like helices around the 3_1_-screw axis with blades consisting of flat ribbons (Table 1, Figure 10). Rb^HH^ from adjacent helices aggregate head-to-tail into ribbon dimers through van der Waals interactions (Figure 10a,d). The nearest environment of the dimer motif is composed of six ester molecules from four different ribbon dimers (Figure 10a). However, the packing of triterpenes in **Me-GE** crystals is not as efficient as in ***R*3-but-OA**. The wide channels surrounded by rings E of molecules related by 6_5_ screw axis are filled with severely disordered solvent molecules. In this area, in ***R*3-but-OA**, only small voids were observed (compare Figure 4a and Figure 10a).

Despite a similar scheme of self-association, the structure of ribbons and their dimers in **Me-GE** and **R3-but-OA** is not identical. First of all, the R-value of (+)17.6° for **Me-GE** (Table S2) indicates a different rotation of molecules within the ribbon in these two structures (compare Figure 4c and Figure 10c). As pointed out by the plus sign, the ester group in **Me-GE** is located on the same side of the ribbon plane as the C11 atom, so it is not directed into the interior of the dimer as in **R3-but-OA**. Changes in the rotation result in the formation of different C-H···O hydrogen bonds within the ribbon in **Me-GE** compared with **R3-but-OA** (Table S3). Secondly, the ribbons of the dimer motif in **Me-GE** are related by 2_1_ screw axis parallel to the ribbon direction. This symmetry element contributes to the mutual displacement of GE ester molecules in both the ribbon direction and triterpene long-axis direction (compare Figure 4a,d and Figure 10a,d).

#### 2.3.2. Crystal Structure and Thermal Analysis of Me-11-oxo-OA

The methyl ester of the 11-oxo OA derivative (Scheme 1) crystallizes as a solvate in the *P*2_1_2_1_2_1_ space group (Table S1). The TGA and DSC thermograms are shown in Figure S7. The asymmetric unit is composed of two triterpenes, A and B, and one 2-propanol molecule (Figure S8). The latter is modeled as disordered over three positions with 0.58, 0.26, and 0.16 occupancy factors.

**Figure 11:**
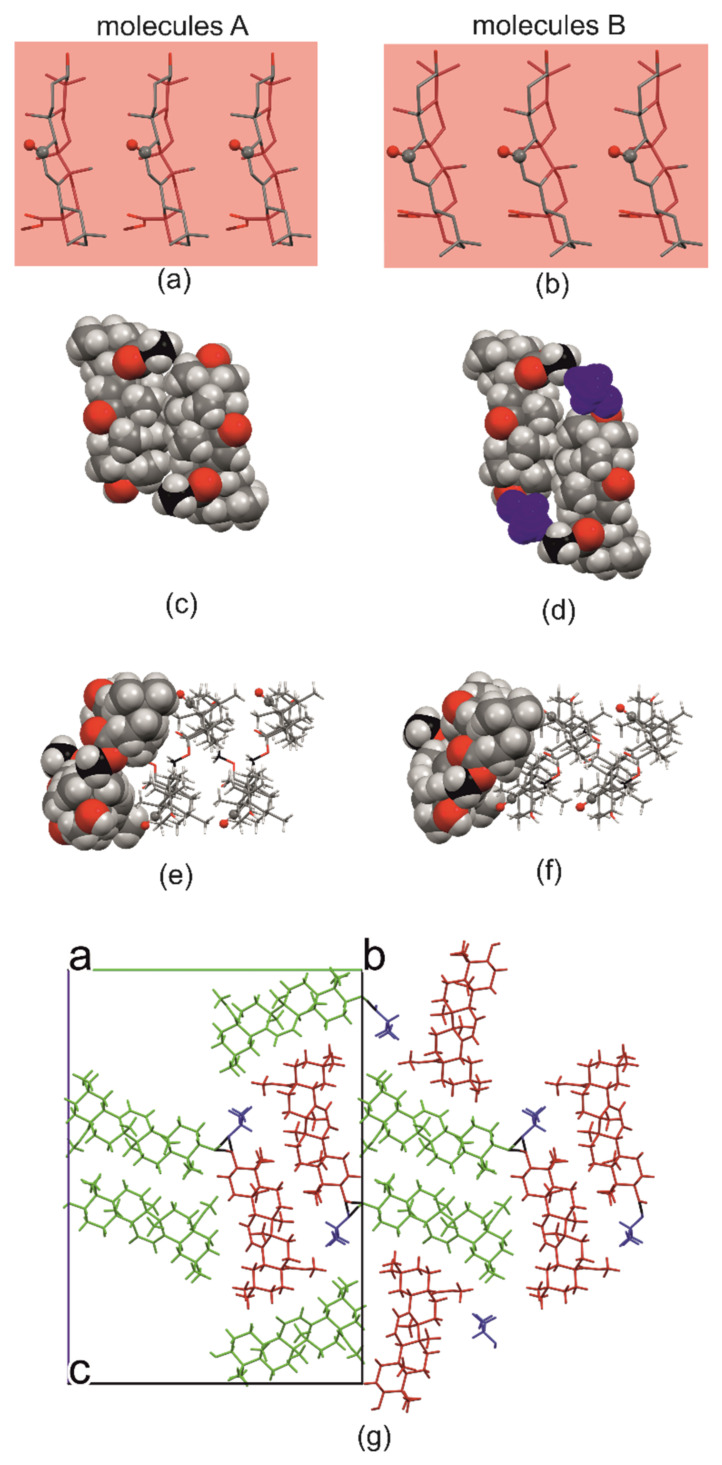
(**a**,**b**) Rb^HH^ ribbons formed in the **11-oxo-Me-OA**: The C11/O11 atoms are shown as balls; the ribbons’ plane is shown in light red; H atoms are omitted for clarity. Dimer shown along ribbons’ direction (**c**,**d**) and triterpene long axis (**e**,**f**). The methyl substituents are colored in black and 2-propanol molecules in violet. (**g**) The nearest surroundings of the dimer motif. The O-H···O hydrogen bonds are shown as black lines. The molecules A are green, B brown, and 2-propanol dark blue.

As in the structures described earlier, the propeller-like helix in **Me-11-oxo-OA** is created by three spirally arranged molecules joined by O-H···O hydrogen bonds (Table 1). However, unlike other structures where propeller blades are identical due to symmetry, the blades in the **Me-11-oxo-OA** differ. Two are formed by triterpenes (molecules A create ribbon A and molecules B—ribbon B) and the third by 2-propanol solvent (Figure 11a,b,g).

Ribbons of the same type from adjacent propeller-like helices aggregate head-to-tail into two symmetry-independent ribbons’ dimers (consisting of AA or BB ribbons), each having a symmetry of 2_1_ screw axis (Figure 11c–g). Due to the different positions of these axes relative to the triterpene skeleton, the construction of dimer motifs varies. In dimer A, the hydrophobic interface between the backbones of the terpenoids of adjacent ribbons is large (Figure 11c). The displacement of molecules in dimer B leads to a significant reduction in van der Waals interactions between ribbons. This shift is compensated by a propanol molecule hydrogen-bonded to an ester, which plays the role of the acceptor. Thus, the solvent molecules became an integral part of ribbon dimers arising from B molecules (Table 1, Figure 11d).

The R values are (−)46.9° and (−)38.8° for ribbons A and B, respectively, indicating that the ester substituent and the C11 atom of ring C are located at different sides of the ribbon (Figure 11a,b,e,f). The R values are similar to those found in the ***P*2_1_-but-OA** crystal structure (−)49.2°, and ribbons in both crystals are supported with the same C-H···O hydrogen bonds (Table S3). As in ***R*3-but-OA**, the ester groups are directed towards the inside of the dimer (compare Figure 4a,d and Figure 11e,f).

### 2.4. Hirshfeld Surface Analysis [49]

**Figure 12:**
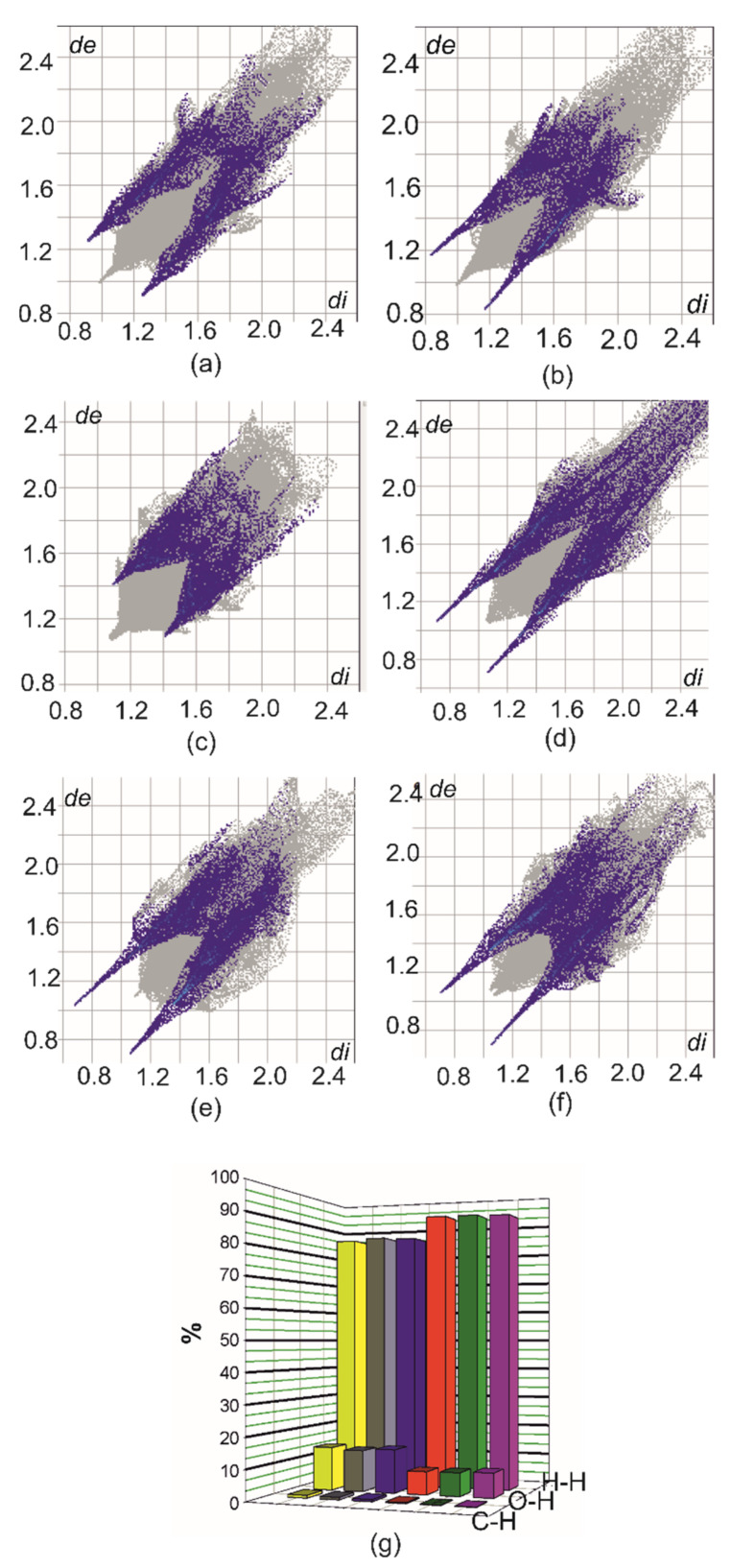
Contribution of O···H intermolecular interactions to the Hirshfeld surface area for molecules (**a**) A and (**b**) B in ***R*3-but-OA**, molecules in (**c**) ***P*2_1_-but-OA**, (**d**) **Me-GE**, and molecules (**e**) A and (**f**) B in **Me-11-oxo-OA**: (**g**) The percentage of H···H, O···H and C···H interactions for (**a**) A (purple) and (**b**) B molecules (green) in ***R*3-but-OA**, molecules in (**c**) ***P*2_1_-but-OA** (red), (**d**) **Me-GE** (dark blue), and molecules (**e**) A (gray) and (**f**) B (yellow) in **Me-11-oxo-OA**. Bars from purple to yellow represent molecules in the order of this caption for fingerprint plots.

Hirshfeld surface analysis allows the quantification of intermolecular interactions. In both polymorphs of OA butyl ester, the percentage contribution of H···H, O···H and C···H contacts are similar, around 92%, 7.5% and 0.1%, respectively (Figure 12g, Table S5). However, as demonstrated with fingerprint plots, these contacts differ (Figure 12a–c and Figure S9). For ***R*3-but-OA** in fingerprint plots, short O···H contacts, around 1.2 Å, are visible as two outside whiskers (Figure 12a,b). These contacts represent O-H···O hydrogen bonds that link molecules into helical chains. Contrarily, no whiskers on the fingerprint plot are observed in the ***P*2_1_-but-OA** (Figure 12c). The shortest O···H interactions, around 1.4 Å, come from C-H···O interactions between the ester carbonyl O28 atom and the C16H_2_ group that supports R^HH^ formation (Table S3).

Compared with ***R*3-but-OA**, the percentage contribution of H···H decreased in favor of O···H contacts in **Me-11-oxo-OA** and **Me-GE** (Figure 12d–g and Figure S9, Table S3). This is due to an additional oxo O atom introduced into the triterpene core and due to the formation, along with the O-H···O hydrogen bonds, of C-H···O interactions, which, similar to the ***P*2_1_-but-OA**, support the formation of R^HH^ motifs (Table S3).

## 3. Materials and Methods

### 3.1. Synthesis and Crystallization

#### 3.1.1. Synthesis

OA derivatives were synthesized according to earlier published procedures [34]. More details about synthesis and the spectral data for the new methyl ester of 11-oxo oleanolic acid are given in Supplementary Materials. During allyl oxidation of acetyloleanolic acid methyl ester, the mixture of methyl ester of 11-oxooleanolic acid and 12-oxoolenolic acid was formed. However, in the crystal structure, only the 11-oxo-OA derivative was observed.

#### 3.1.2. Crystallization

All single crystals used for X-ray analysis were obtained by slow evaporation at room temperature. Solvents were purchased from commercial suppliers and used without further purification.

Single-crystal ***R*3-but-OA**: About 10 mg of OA butyl ester was dissolved in DMF (100 μL). The needle-like crystals appeared after 5 d. The solid form, i.e., polymorph or amorphous content, was not controlled in starting material.

Single-crystal **Me-11-oxo-OA**: About 10 mg of the sample (the mixture of methyl ester of 11-oxo- and 12-oxo-OA) was dissolved in 2-propanol (100 μL). The plate-like crystals appeared after 5 d.

#### 3.1.3. Screening for Polymorphs

Samples of about 100 mg of the OA butyl ester (the monoclinic polymorph *P*2_1_-but-OA) were dissolved in ethyl acetate, ethanol, 2-propanol, and dimethylsulfoxide and left to crystallize at ambient conditions.

#### 3.1.4. Slurry Experiments

For suspension preparation in ethanol, the ***R*3-but-OA** phase was used. The slurries were harvested for powder diffraction experiments after one week and two weeks.

### 3.2. Characterization Techniques

#### 3.2.1. X-ray Diffraction

Single-crystal X-ray data were collected at 130 K with an Oxford Diffraction SuperNova Dual diffractometer using high-flux microfocus Nova CuKα radiation (λ = 1.5418 Å) and processed with the CrysAlis Pro, version 1.171.38.43 software [50]. The structures were solved by direct methods with SIR2014 [51] and refined by full-matrix least squares on F^2^ with SHELX2014 [52]. The absolute structure of the studied compounds is based on the Flack parameter and corresponds to the known absolute configuration of OA.

All non-H atoms were refined anisotropically. The C-bound H atoms were included in calculated positions and refined as riding on their corresponding carbon atoms with Uiso(H) = 1.2Ueq(CH, CH_2_) or 1.5Ueq (CH_3_). The H atoms of O–H groups were located in difference electron-density maps and constrained to an ideal position with O–H = 0.84 Å and Uiso(H) = 1.5Ueq(O). The ester group of one symmetry-independent molecule in ***R*3-but-OA** and 2-propanol molecule in **Me-11-oxo-OA** is disordered. DFIX and EADP commands were used to restrain geometry and atomic displacement parameters for these fragments. Details on structure refinements are presented in Table S1. The geometry of hydrogen bonds is shown in Table 1 and Table S3. The numbering of atoms is given in Figure 3.

#### 3.2.2. Powder Diffraction Experiments

Powder X-ray data were collected on a Bruker AXS D2 Phaser diffractometer (Bruker, Karlsruhe, Germany) with Cu Kα radiation. Before X-ray diffraction experiments, samples were ground with a mortar and pestle. Steel holders with 25 mm diameter and 1 mm depth were used for measurements. Scans were collected with a 2θ range between 5–35°, a step size of 0.02°, a counting rate of 2 s/step, and operating conditions of 30 kV and 10 mA. The samples were spinning at 30 rpm. A 1 mm slit module was used during measurements.

#### 3.2.3. Differential Scanning Calorimetry (DSC)

Differential scanning calorimetry thermograms were collected with DSC 214 Polyma (NETZSCH, Selb, Germany). Samples were closed in aluminum pans with the pierced cover. For the DSC experiments followed by PXRD, open pans were used. The nitrogen atmosphere (30 mL/min) was maintained during measurements. The scans were made with a heating rate of 5 K/min.

For the first DSC measurements (6.46 mg of an amorphous sample of butyl OA ester), two heating (to 170 °C) and one cooling (to 7 °C) cycle were performed. The second experiment for an amorphous sample was set as follows: 5.36 mg of butyl OA ester was heated to 70 °C and kept at this temperature for 20 min, then cooled, and the PXRD pattern was recorded. Then, it was heated to 138 °C, held at this temperature for 20 min, cooled, and the PXRD pattern was recorded.

#### 3.2.4. Thermogravimetry (TGA) 

Thermogravimetric analysis was performed using a TG 209 F3 Tarsus instrument (NETZSCH, Selb, Germany). The open corundum crucible was used for measurements. The analyzed samples were heated at a 5 K/min rate under a nitrogen atmosphere (30 mL/min).

#### 3.2.5. Temperature Measurement by the Boetius Method

The visual changes in the samples during heating and cooling were recorded with the Boetius apparatus (VEB Analytik Dresden, Rathenow, Germany), employed with a thermometer, optical microscope, and camera (Delta Optical, DLT-Cam PRO 6.3 MP, Nowe Osiny, Poland). The heating rate was about 10 °C per minute. The sample was placed between two glass plates on the heating table during measurements.

### 3.3. Theoretical Calculations

The Hirshfeld surfaces and fingerprint plots were calculated using CrystalExplorer21, Version 21.5 software [49]. Surfaces were computed with a high (standard) resolution with d_norm_ property plotted and isovalue 0.5. The B3LYP method and 6-31G (d,p) basis set implemented in the CrystalExplorer21 program were utilized for the intermolecular interaction energies’ estimation [42]. The total energy (E_tot_) was decomposed into electrostatic (E_ele_), polarization (E_pol_), dispersive (E_disp_), and repulsion (E_rep_) components. The scale factors used for E_tot_ calculation are 1.057, 0.740, 0.871, and 0.618 for E_ele_, E_pol_, E_disp_, and E_rep_, respectively. Energy frameworks [53] of the total, dispersion, and electrostatic intermolecular interaction energies were visualized with CrystalExplorer21 software. The tubes are adjusted to the scale factor of 80 with a cutoff of 5 kJ/ mol.

Intermolecular interactions’ energy calculations were also performed with PIXEL program [43] using the Mercury interface [54]. Energy-vector models were generated using *process*Pixel [55]. Molecular electron densities were obtained using *Gaussian 09* [40] at the MP2/6-31G** level of theory. The molecule energy was also calculated with *Gaussian 09* [40] at the B3LYP/6-31G (d,p) level of theory.

In addition to CrystalExplorer (CE-B3LYP) and PIXEL, the Psi4 program [44] was applied for SAPT and supramolecular interaction energies (IEs) calculations. SAPT0 [56,57,58] computations were carried out using the jun-cc-pVDZ basis set [59]. For selected complexes, additional methods were also applied SAPT0-D3M(BJ)/jun-cc-pVDZ [60], as well as the supramolecular approach for IEs at the ωB97M-V/def2-TZVPD level of theory [45] with counterpoise corrections for the effects of basis set superposition error [46].

Graphical representations of noncovalent interactions (NCI plots) were obtained using programs MultiWfn 3.7 [61] and VMD 1.9.4a53 [62].

The crystal structures established at 130 K were used for all energy calculations. The C and H atoms of disordered butyl substituent with minor occupancies factor were removed from the ***R*3-but-OA** structure.

## 4. Conclusions

New structures of esters of oleanolic acid and its 11-oxo derivative presented in this manuscript manifest alternative modes of molecular aggregation to the already known layered supramolecular architectures of OA and 11-oxo OA esters. In studied crystals, the assembly of molecules leads to the formation of hydrogen-bonded helices. This 1D motif does not occur in layered structures based mainly on van der Waals interactions [9,34].

Intermolecular interaction energy calculations of ***R*3-but-OA** (helical structure) and ***P*2_1_-but-OA** (layered structure) show that ribbons formed by mainly van der Waals forces are the most stable motifs in both structures. However, the head-to-tail ribbon dimers found in the metastable ***R*3-but-OA** form are less energetically stabilized than similar motifs distinguished as a part of the layer in the thermodynamically preferred ***P*2_1_-but-OA** polymorph. In addition, the overall 3D architecture of ***R*3-but-OA** lowers the packing coefficient; hence, small voids in a crystal structure are observed. The helical structure of GE methyl ester corroborates that aggregation of O-H···O hydrogen-bonded chains reduces the crystal packing efficiency, i.e., solvent molecules are required for crystal formation.

Although hydrogen bonding is believed to stabilize crystal structures, our studies showed that, in the case of OA and GE esters, the formation of directional hydrogen bonds is not only energetically less favorable but also disturbs efficient 3D packing.

## Figures and Tables

**Table 1:** Hydrogen-bond geometry (A, °). Note: Calculated based on: * Beseda [35].

D—H···A	D—H	H···A	D···A	D—H···A
***R*3-but-OA**				
O3A—H3A1···O3A ^i^	0.84	2.30	3.115 (4)	164
O3B—H3B1···O3B ^ii^	0.84	2.06	2.878 (4)	163
**Me-11-oxo-OA**				
O3A—H3OA···O1S	0.84	1.86	2.696 (4)	173
O3B—H3OB···O3A ^iii^	0.84	1.90	2.708 (4)	160
O1S—H1S···O3B	0.84	1.90	2.735 (4)	174
Me-GE *				
O1-H39···O1 ^iv^	0.84	1.93	2.745	162

Symmetry codes: (i) −y + 2/3, x – y + 1/3, z + 1/3; (ii) −y + 4/3, x – y + 2/3, z − 1/3; (iii) x − 1, y, z; (iv) y − x, 1 − x, 1/3 + z. S—solvent molecule (2-propanol).

## Data Availability

Row data is available directly from the corresponding authors upon reasonable request.

## Supplementary Materials

The following supporting information can be downloaded at: https://www.mdpi.com/article/10.3390/ijms24076572/s1.

## Appendix A

The interaction energy (IE) values discussed in the text and shown in the figures are from CrystalExplorer calculation [42], except for O-H···O hydrogen-bonded helices, for which results from ωB97M-V/def2-TZVPD are given. All energy values are provided in Table S4.

To verify the values of the individual components of the total interaction energy (electrostatics, exchange, induction, and dispersion) calculated by CE-B3LYP and PIXEL, the Symmetry-Adapted Perturbation Theory (SAPT) approach was used. It was demonstrated in [58,63] that SAPT0 with truncated jun-cc-pVDZ basis sets performs well in calculations of IE because of a favorable cancellation of errors.

Comparing the results obtained by CE-B3LYP, PIXEL and SAPT0, interaction energies calculated by the latter were slightly lower (less binding) than IEs calculated by CE-B3LYP or PIXEL. A better agreement of interaction energies is observed for SAPT0 and PIXEL results. Additionally, dispersion and repulsion (exchange) components in studied structures were usually underestimated in SAPT0. Nevertheless, the methods consistently indicate the increasing or decreasing ratio of the dispersion in the interaction energy of individual complexes. The methods are also consistent regarding the value of E_ele_ in total energy, showing its dominant character among attractive interactions in complexes with hydrogen bonds.

The CE-B3LYP method uses the D2 empirical corrections to calculate dispersion terms; moreover, E_ele_ and E_rep_ are calculated similarly to SAPT expansion for electrostatic and exchange energies [42]. Thus, in the case of several structural motifs, the test of the agreement of the calculated interaction energies (E_tot_) and the dispersion component (E_dis_) values between CE-B3LYP and the SAPT0-D3M(BJ) method with the jun-cc-pVDZ basis set was performed. Replacing dispersion terms from SAPT0 expansion with the empirical parameters in SAPT0-D3M(BJ) reduced the differences between the calculated interaction energies by SAPT0-D3M(BJ) and CE-B3LYP and PIXEL. SAPT0-D3M(BJ) predicts even stronger stabilization for motifs with a predominance of dispersion interactions. The stabilization energy for complexes with hydrogen bonds is slightly lower than for the CE-B3LYP and PIXEL methods.

Because of discrepancies in the IE results obtained by the above methods, a supramolecular approach was used to calculate interaction energies for selected structural motifs. The ωB97M-V functional, which gave good results in studies of various types of noncovalent interactions (NCI), was used [45,46,64,65]. The IEs obtained at the ωB97M-V/def2-TZVPD level agree very well (within 5 kJ/mol) with the results of CE-B3LYP and PIXEL for dispersion-predominant complexes. Slightly larger differences (within 8 kJ/mol) are observed for hydrogen-bonded complexes, and the error signs for these complexes indicate overbinding by CE-B3LYP and PIXEL.
