# Supplementary material for: Polymorphism of Butyl Ester of Oleanolic Acid—The Dominance of Dispersive Interactions over Electrostatic
Source: Int J Mol Sci. 2023 Mar 31;24(7):6572. doi: 10.3390/ijms24076572 (PMC10095383; doi:10.3390/ijms24076572)
Supplement: Supplementary file 1 [file ijms-24-06572-s001.zip › IJMS_SI.pdf]

Supporting information for:

Polymorphism of butyl ester of oleanolic acid – the dominance of dispersive interactions over electrostatic

Dominik Langer[1], Barbara Wicher[1*], Zbigniew Dutkiewicz[1], Wioletta Bendzinska-Berus[1], Barbara Bednarczyk-Cwynar[2] and Ewa Tykarska[1*]

[1]Department of Chemical Technology of Drugs, Poznan University of Medical Sciences, Grunwaldzka 6, Poznan, 60-780, Poland
[2]Department of Organic Chemistry, Poznan University of Medical Sciences, Grunwaldzka 6, Poznan, 60-780, Poland
*Correspondence email: bwicher@ump.edu.pl; etykarsk@ump.edu.pl

Synthesis

For reactions in anhydrous conditions, the solvents were purified and dried by using common procedures.

Thin layer chromatographic (TLC) analysis (reactions progress and the level of compounds purity) was conducted on HPTLC aluminum sheets covered with silica gel 60 F245. Benzene ethyl acetate (1:1, 2:1, 4:1, or 9:1, v:v) was used as an eluent. The chromatograms were visualized by spraying with 10% ethanolic solution of sulfuric acid and heating the plates at about 110 °C for a few minutes.

Column chromatography was performed using silica gel 60 (0.063–0.200 mm, 70–230 mesh) and methylene chloride and acetone (9:1, v:v) as an eluent.

Melting points were measured with a Büchi apparatus in an open capillary and are uncorrected.

NMR spectra for hydrogen atoms (^{1}H) and carbon atoms (^{13}C) were recorded in deuterated chloroform solutions ($CDCl_3$), at frequencies of 600 and 150 MHz, respectively, with tetramethylsilane (TMS) as an internal standard, with the application of Varian Gemini 300 VT spectrometer. The values of chemical shifts are given in δ, for both the ^{1}H and the ^{13}C spectra, with an accuracy of ±0.01 ppm. J-values are presented with an accuracy of ±0.1 Hz. The standard scheme of describing the multiplicities of signals in ^{1}H NMR spectra was used: s – singlet, d – doublet, t – triplet, dt – doublet of triplets, dd – doublet of doublets, td – triplet of doublets and are listed below.

MS spectra were recorded using an AMD 402 spectrometer with electroionization.

Synthesis of **Me-11-oxo-OA** was performed with four steps:

1. Oleanolic acid methylation:

Oleanolic acid (starting material): mol. formula: $C_{30}H_{48}O_3$; mol. mass: 456.360346; m.p.: 308 – 310 °C (EtOH); spectral data in agreement with those from: Ahmad V. U.; Atta-ur-Rahman. Handbook of Natural Products Data. Volume 2. Pentacyclic Triterpenoids; Elsevier: Amsterdam – London – New York – Tokio, 1994; 1 –509.

K_2CO_3 (2,5 mmol) was added to a stirred and heated in 80 °C saturated solution of oleanolic acid (2 mmol) in dried DMF. After 30 minutes of further heating and stirring, methyl chloride (2.5 mmol) was added dropwise. The resulting mixture was heated and stirred for total consumption of starting material (TLC control), cooled, and poured into water (about 6-fold volume) slightly acidified with HCl. The obtained precipitate of methyl ester of oleanolic acid was filtered off, washed with water, dried, and crystallized.

Methyl ester of oleanolic acid (= methyl oleanolate): **mol. formula**: $C_{31}H_{50}O_3$; **mol. mass**: 470.375996; **yield**: 96%; **mp.**: 199 – 201°C (EtOH); lit.: 199 – 201 °C; spectral data in agreement with those from: Bednarczyk-Cwynar, B.; Ruszkowski, P.; Bobkiewicz-Kozlowska, T.; Zaprutko, L.. Oleanolic Acid A-lactams Inhibit the Growth of HeLa, KB, MCF-7 and Hep-G2 Cancer Cell Lines at Micromolar Concentrations. *Anti. Cancer Agents Med. Chem.* **2016**, 16, 579–592. doi:10.2174/1871520615666150907095756.

.

2. Acetylation of oleanolic acid methyl ester:

A saturated solution of methyl oleanolate (2 mmol) in acetic anhydride was heated under reflux for 15 min. Next, it was cooled and poured into the water with ice (6-fold volume). The precipitate was filtered off, washed with water, and dried. The resulted residue was filtered off, washed with water, dried, and crystallized.

Methyl ester of acetyloleanolic acid (= methyl acetyloleanolate): **mol. formula**: $C_{33}H_{52}O_4$; **mol. mass**: 512.386560; **yield**: 96.2 %; **mp.**: 226 – 227 °C (EtOH); lit.: 229 – 230 °C; spectral data in agreement with those from: Okamoto, I.; Takeya; T.; Kagawa, Y.; Kotani, E. Iron(III)Picolinate-Induced Oxygenation and Subsequent Rearrangement of Triterpenoid Derivatives with Hydrogen Peroxide. *Chem. Pharm. Bull.* **2000**, 48, 120–125. doi:10.1248/cpb.48.120.

.

3. Allyl oxidation of acetyloleanolic acid methyl ester:

Sodium dichromate dihydrate (4 mmol) was added to a saturated solution of methyl acetyloleanolate, (2 mmol) in acetic acid and heated at 70 °C to the total consumption of starting material (TLC control). The mixture reaction was cooled, a small amount of isopropyl alcohol was added, and the mixture was filtered off. The filtrate was poured into water (6-fold volume), and the obtained precipitate was filtered off, washed with water, dried, and evaporated to dryness. The resulted residue was subjected to column chromatography separation. The received crude product was crystallized.

Methyl ester of 11-oxoacetyloleanolic acid (= methyl 11-oxoacetyloleanolate): **mol. formula**: $C_{33}H_{50}O_5$; **mol. mass**: 526.365825; **yield**: 88.0 %; **mp.** 248 – 250 °C (EtOH); **^{1}H NMR**, δ: 5.64 (1H, s, C_{12}-H); 4.51 (1H, dd, *J* = 4.9 and 11.6 Hz, C_3-H_α); 3.63 (3H, s, -COO-$\underline{CH_3}$); 3.00 (1H, dd, *J* = 4.8 and 14.0 Hz, C_{18}-H_β); 2.83 (1H, dt, *J* = 3.5 and 13.6 Hz, C_{19}-H_a), 2.34 (1H, s, C_9-H_α), 2.05 (3H, s, $\underline{CH_3}$-COO-); 2.03 (1H, td, *J* = 13.7, 4.1 Hz, C_{19}-H_b); 1.36, 1.13, 0.93, 0.93, 0.91, 0.87 x 2 (6 x 3H + 1 x 6H, singlets, 7 x CH_3 groups of oleanane skeleton); **^{13}C NMR**, δ: 200.09 (C_q, C-11); 177.39 (C_q, C-28, -$\underline{CO}$O-CH_3); 170.89 (C_q, CH_3-$\underline{CO}$O-); 168.60 (C_q, C-13); 127.78 (CH, C-12); 80.52 (CH, C-3); 61.59 (CH, C-9); 51.81 (CH_3, -COO-$\underline{CH_3}$); 46.12 (C_q, C-17); 21.23 (CH_3, $\underline{CH_3}$-COO-); **MS/EI** (m/z): 526.6 (34.2 %, $M^{+\bullet}$).

Methyl ester of 12-oxoacetyloleanolic acid (= methyl 12-oxoacetyloleanolate): **mol. formula**: $C_{33}H_{50}O_5$; **mol. mass**: 526.365825; **^{1}H NMR**, δ: 4.47 (1H, dd, *J* = 4.8 and 11.8 Hz, C_3-H_α); 3.68 (3H, s, -COO-$\underline{CH_3}$), 2.77 (1H, dt, *J* = 4.3 and 13.4 Hz, C_{18}-H_β); 2.61 (1H, d, *J* = 4.3 Hz, C_{13}-H); 2.24 (1H, dd, *J* = 5.2 and 17.0 Hz, C_{11}-H_a); 2.13 (1H, dd, *J* = 2.4 and 13.5 Hz, C_{11}-H_b); 0.97 x 2, 0.96, 0.90, 0.86; 0.80, 0.78 (5 x 3H + 2 x 6H, singlets, 7 x CH_3 groups of oleanane skeleton); **^{13}C NMR** (101 MHz, $CDCl_3$) δ 211.52 (C_q, C-12); 178.28 (C_q, C-28, -COO-$\underline{CH_3}$); 170.79 (C_q, CH_3-$\underline{CO}$O-); 80.32 (CH, C-3); 51.74 (C_q, C-13); 51.72 (CH_3, -COO-$\underline{CH_3}$); 47.25 (C_q, C-17); 21.20 (CH_3, $\underline{CH_3}$-COO-); **MS/EI** (m/z): 528.7 (11.8 %, $M^{+\bullet}$).

4. Alkaline hydrolysis of acetyloleanolic acid methyl ester:

A saturated solution of methyl 11-oxoacetyloleanolate (2 mmol) in a 5% ethanolic solution of NaOH was heated under reflux for 15 min. Next, it was cooled, poured into water (6-fold volume) and slightly acidified with HCl. The precipitate was filtered off, washed with water, dried and crystallized.

Methyl ester of 11-oxooleanolic acid (= methyl 11-oxooleanolate): mol. formula: $C_{31}H_{48}O_4$; **mol. mass**: 484.355260; **yield**: 96.2 %; **mp.** 195 – 196 °C (C_6H_6); **^{1}H NMR**, δ: 5.64 (1H, s, C_{12}-H); 3.63 (3H, s, -COO-$\underline{CH_3}$); 3.22 (1H, dd, *J* = 10.6 and 5.6 Hz, C_3-H_α); 3.00 (1H, dd, *J* = 13.8 and 4.2 Hz, C_{18}-H_β); 2.83 (1H, dt, *J* = 13.5 and 3.4 Hz, C_{19}-H_a); 2.32 (1H, s, C_9-H_α); 2.05 (1H, td, *J* = 13.9 and 4.3 Hz, C_{19}-H_b); 1.36; 1.10; 0.99; 0.93; 0.93; 0.91; 0.79 (7 x 3H, singlets, 7 x CH_3 groups of oleanane skeleton); **^{13}C NMR**, δ: 200.35 (C_q, C-11); 177.46 (C_q, C-28, -$\underline{CO}$O-CH_3); 168.68 (C_q, C-13); 127.81 (CH, C-12); 78.67 (CH, C-3); 61.71 (CH, C-9); 46.13 (C_q, C-17); **MS / EI** (m/z): 484.6 (43.8 %, $M^{+\bullet}$).

Methyl ester of 12-oxooleanolic acid (= methyl 12-oxooleanolate): mol. formula: $C_{31}H_{50}O_4$; **mol. mass**: 486.370910; **^{1}H NMR**, δ: 3.68 (3H, s, -COO-$\underline{CH_3}$); 3.20 (1H, dd, *J* = 9.5 and 5.4 Hz, C_3-H_α); 2.82 (1H, dt, *J* = 13.5 and 3.4 Hz, C_{18}-H_β); 2.61 (1H, d, *J* = 4.1 Hz, C_{13}-H); 2.24 (1H, dd, *J* = 16.8 and 5.3 Hz, C_{11}-H_a); 2.15 (1H, dd, *J* = 17.4 and 4.4 Hz, C_{11}-H_b); 0.97; 0.96; 0.85; 0.78; 0.71; 0.70; 0.67 (7 x 3H, singlets, 7 x CH_3 groups of oleanane skeleton); **^{13}C NMR**, δ: 211.87 (C_q, C-12); 178.33 (C_q, C-28, -$\underline{CO}$O-CH_3); 78.54 (CH, C-3); 51.75 (C_q, C-13); 51.75 (CH_3, -COO-$\underline{CH_3}$); 47.27 (C_q, C-17); **MS/EI** (m/z): 486.7 (22.9 %, $M^{+\bullet}$).

Figures

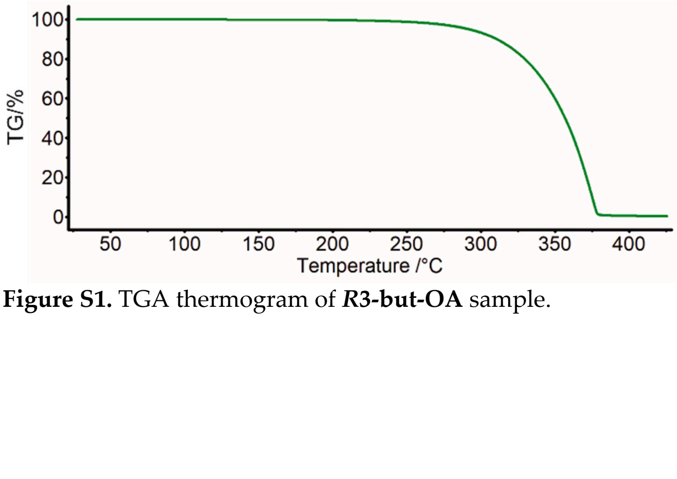

Figure S1. TGA thermogram of ***R*3-but-OA** sample.

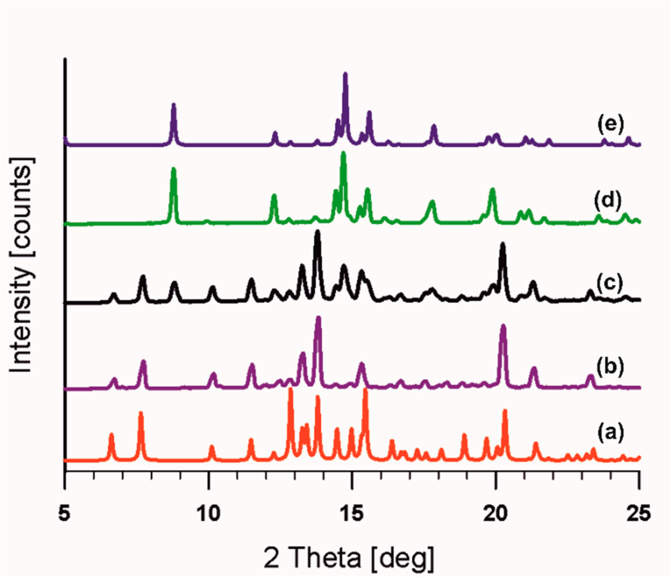

Figure S2. Powder diffractograms simulated based on the crystal structure of a) ***R*3-but-OA** and e) ***P*2₁-but-OA** and registered during slurry experiments: b) starting ***R*3-but-OA** phase, c) after one week (mixture of ***R*3-but-OA** and ***P*2₁-but-OA**, d) after two weeks, pure ***P*2₁-but-OA**.

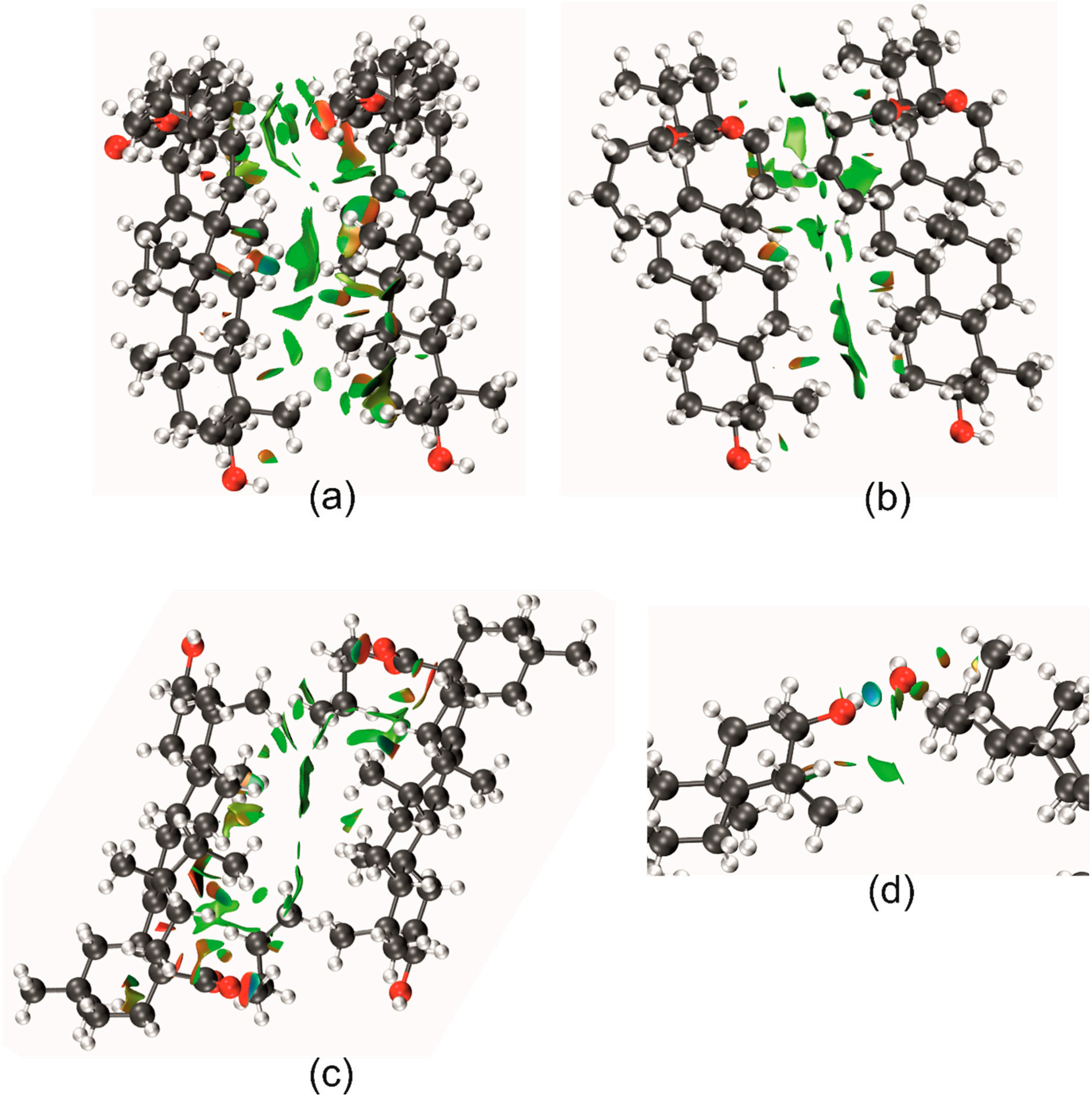

Figure S3 Non-covalent interaction (NCI) plots for a) ribbon in ***P*2_1-but-OA**, b) ribbon B in ***R*3-but-OA**, c) dimer in ***R*3-but-OA**, d) hydrogen-bonded molecules B in ***R*3-but-OA.** The RDG isosurface value is 0.5, and the color range is -0.03 – 0.02; attractive interactions from blue (H-bonds) to green (van der Waals), repulsive from yellow to red (steric).

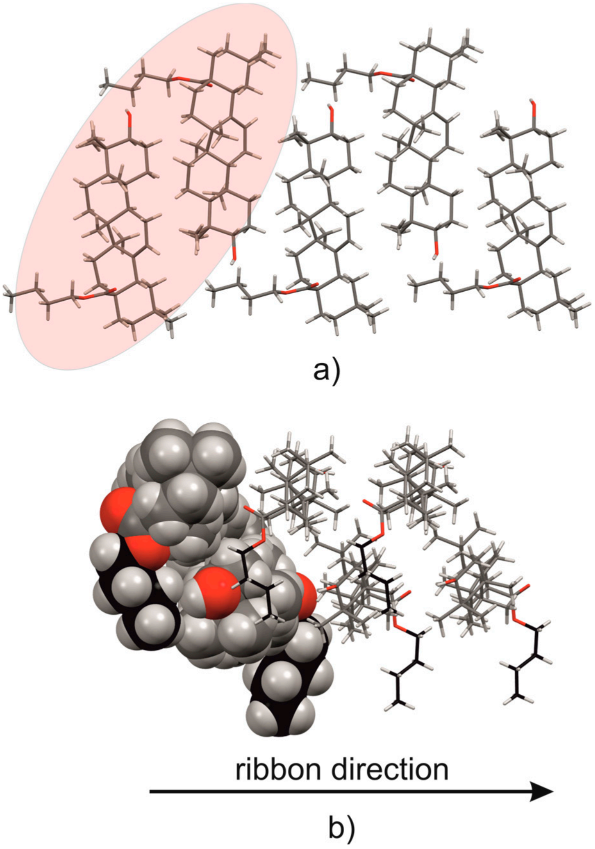

Figure S4. Layer viewed along ribbon direction (a) and ribbons' dimer (b) distinguished in ***P*2₁-but-OA**. In (a) dimer is shaded with a red ellipse. In (b) is shown along the triterpene long axis, and the C-atoms of the butyl group are colored black. Figures prepared based on crystal structures published by Langer [34].

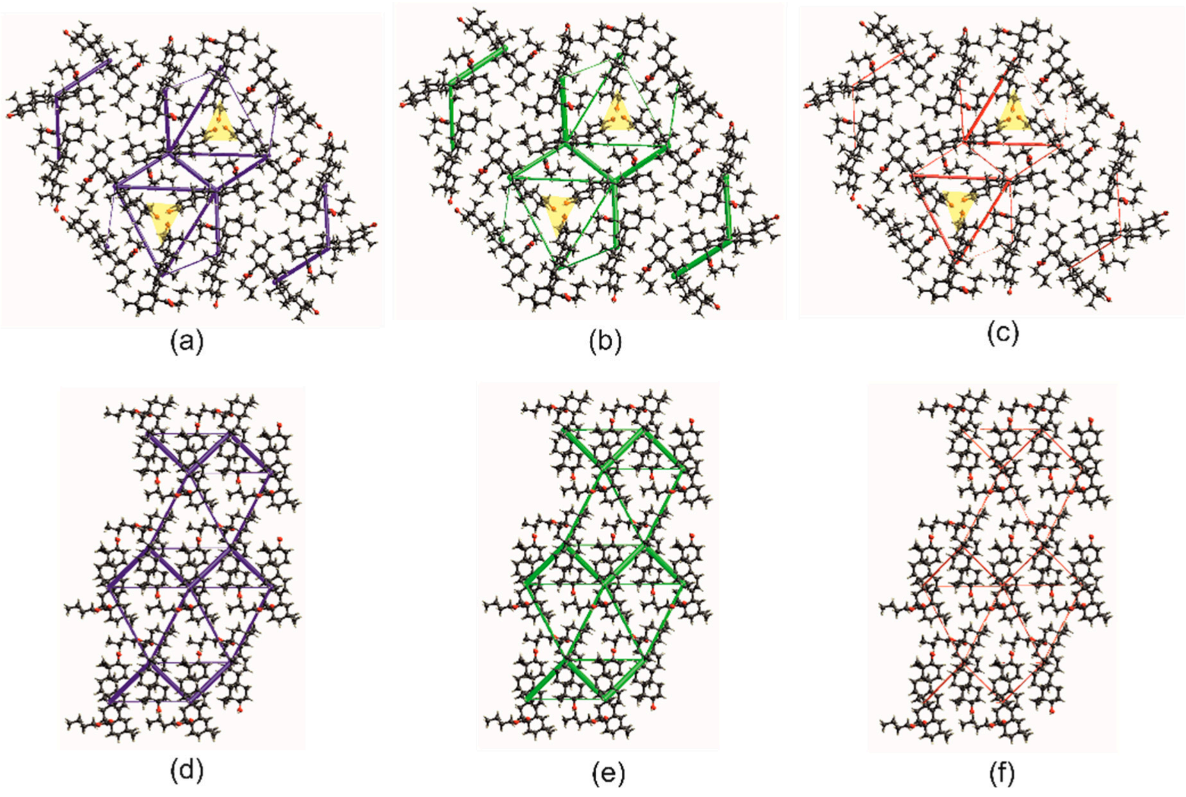

Figure S5. Energy frameworks for ***R*3-but-OA** (a-c) and ***P*2_1-but-OA** (d-f). Total (blue), dispersion (green) and electrostatic (red) interaction energies are shown as cylindrical tubes with diameters proportional to the magnitude of the energies. Structures are shown along the ribbons' direction, so the strongest dispersion interactions are not visible. In a-c, hydrogen-bonded OH groups are marked with yellow triangles.

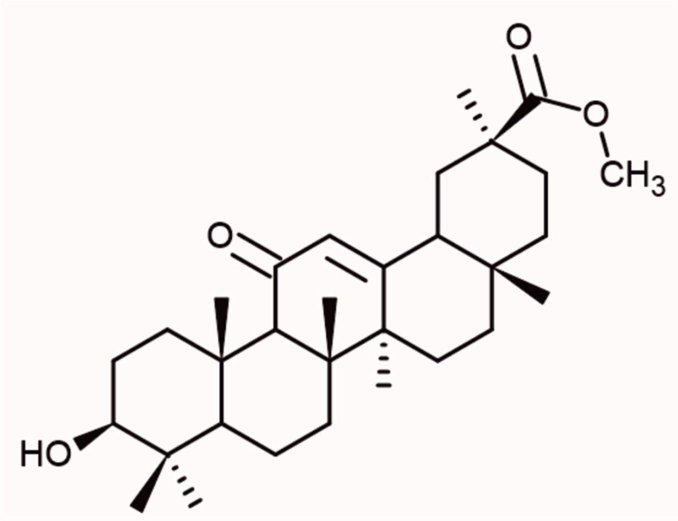

Figure S6. Scheme of **Me-GE**.

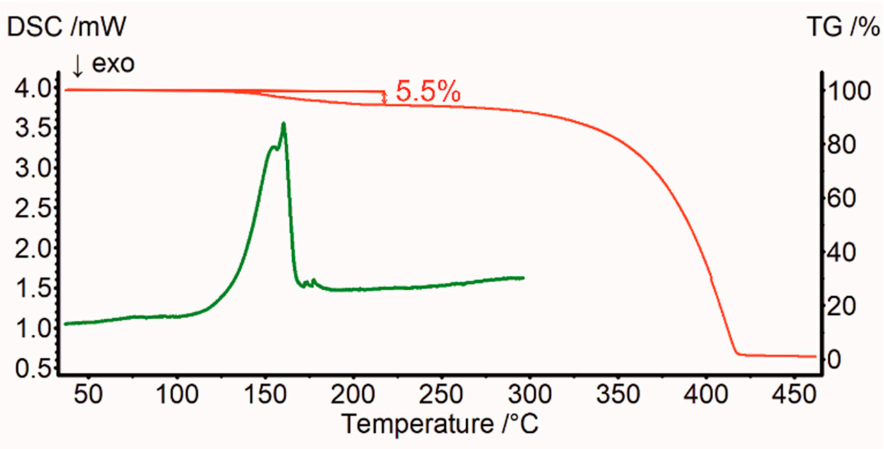

Figure S7. TGA (red) and DSC (green) thermograms of **Me-11-oxo-OA**. The sample of **Me-11-oxo-OA** was subjected to thermogravimetric (TGA) and differential scanning calorimetry (DSC) experiments. The TGA thermogram shows 5.5 % of the mass loss up to 225 °C. It is related to solvent evacuation from the crystal structure. The theoretical mass loss for 2-propanol removal is 5.8 %. The weight change is accompanied by an endotherm at DSC. However, there are no other endothermic events; thus, most likely, along with solvent evaporation, the OA methyl ester is melting.

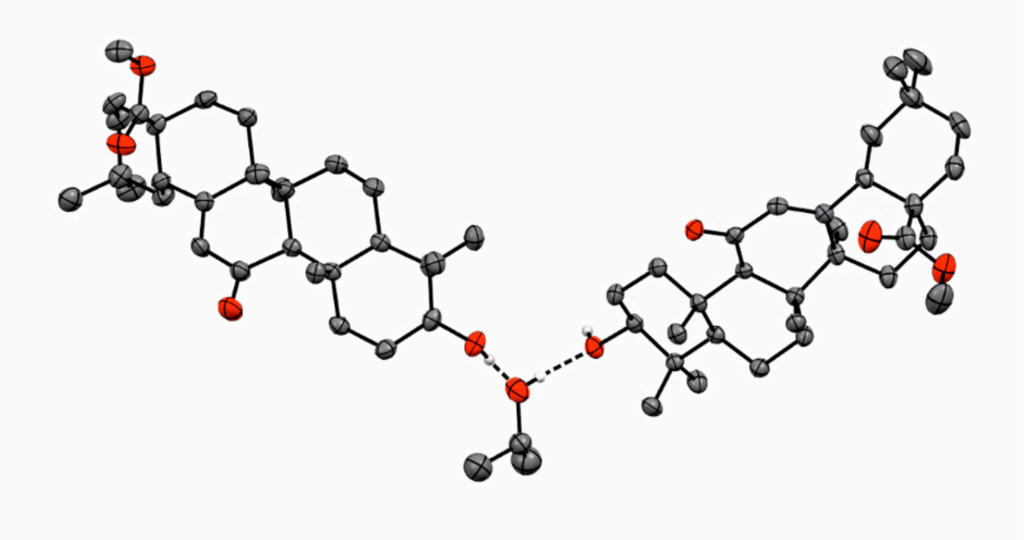

Figure S8. ORTEP representation of an asymmetric unit of **Me-11-oxo-OA**. Except for these from O-H group, the H atoms are omitted for clarity. Displacement ellipsoids are at a 50% probability level. Only the major position of the disordered 2-propanol molecule is shown.

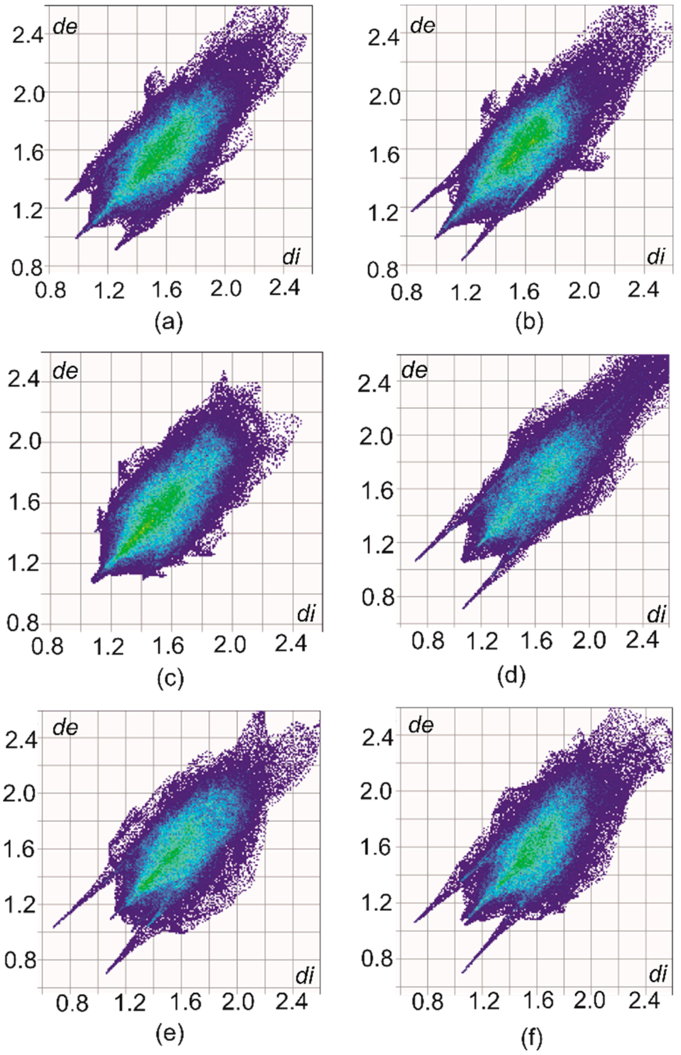

Figure S9. Contribution of intermolecular interactions to the Hirshfeld surface area for molecules a) A and b) B in ***R*3-but-OA**, molecules in c) ***P*2_1-but-OA**, d) **Me-GE**, and molecules e) A and f) B in **Me-11-oxo-OA**.

Tables

Table S1. Experimental details

Experiments were carried out at 130 K with Cu Ka radiation using a SuperNova, Dual, Cu at zero, Atlas. Absorption was corrected by multi-scan methods, CrysAlis PRO 1.171.38.34a [50] Empirical absorption correction using spherical harmonics, implemented in SCALE3 ABSPACK scaling algorithm. H-atom parameters were constrained.

	***R*3-but-OA**	**Me-11-oxo-OA**
Crystal data		
Chemical formula	$C_{34}H_{56}O_3$	$2(C_{31}H_{48}O_4)\cdot(C_3H_8O)$
M_r	512.78	1029.48
Crystal system, space group	Trigonal, *R*3:*H*	Orthorhombic, $P2_12_12_1$
Temperature (K)	130	130
a, *b*, *c* (Å)	46.218 (2), 46.218 (2), 7.3261 (4)	6.7512 (3), 25.119 (1), 35.394 (2)
α, β, γ (°)	90, 90, 120	90, 90, 90
V ($Å^3$)	13552.5 (12)	6002.2 (5)
Z	18	4
μ (mm^{-1})	0.53	0.58
Crystal size (mm)	0.20 × 0.03 × 0.03	0.60 × 0.05 × 0.05
Data collection		
T_{min}, T_{max}	0.789, 1.000	0.698, 1.000
No. of measured, independent and observed [$I > 2\sigma(I)$] reflections	13137, 6131, 4444	37061, 10539, 8674
R_{int}	0.052	0.062
θ_{max} (°)	54.2	66.6
$(\sin \theta/\lambda)_{max}$ ($Å^{-1}$)	0.526	0.595
Refinement		
$R[F^2 > 2\sigma(F^2)]$, $wR(F^2)$, S	0.053, 0.109, 1.01	0.054, 0.148, 1.05
No. of reflections	6131	10539
No. of parameters	678	707
No. of restraints	9	65
$\Delta\rho_{max}$, $\Delta\rho_{min}$ (e $Å^{-3}$)	0.20, −0.20	0.77, −0.30
Absolute structure	Flack x determined using 1370 quotients [(I+)-(I-)]/[(I+)+(I-)]	Flack x determined using 3107 quotients [(I+)-(I-)]/[(I+)+(I-)]
Absolute structure parameter	0.0 (3)	0.10 (13)
CCDC number	2238226	2238227

Computer programs: SIR2004 [51], Mercury [47], CrysAlis PRO 1.171.38.43 [50], SHELXL2014 [52].

Table S2. R values (°). R means rotation of molecules within the ribbon

Note: Calculated based on: * Langer [34]; ** Beseda [35].

	R		R
**R3-but-OA (A)**	(-) 2.8	**$P2_1$-but-OA***	(-) 49.2
**R3-but-OA (B)**	(-) 5.0	**Me-GE****	(+) 17.6
**Me-11-oxo-OA (A)**	(-) 46.9		
**Me-11-oxo-OA (B)**	(-) 38.8		

Table S3. Geometry od C-H···O interactions (A, °).

Note: Calculated based on: * Langer [34]; ** Beseda [35].

D—H···*A*	*D*—H	H···*A*	*D*···*A*	*D*—H···*A*
***R*3-but-OA (A)**				
C34A-H34G···O28A^{i}	0.98	2.58	3.48 (1)	153
**Me-11-oxo-OA**				
C16A-H16A···O28A^{ii}	0.99	2.48	3.445 (5)	164
C16B-H16D···O28B^{i}	0.99	2.50	3.378 (5)	148
**Me-GE****				
C7-H10···O2^{iii}	0.99	2.64	3.62	169
$P2_1$*–but-OA				
C16B-H16D···O28B^{iv}	0.99	2.60	3.573 (2)	167

Symmetry codes: (i) x, y -1+z; (ii) -1+x, y, z; (iii) x, y, -1+z; (iv) 1+x, y,z

Table S4. Interactions energy calculated with CrystalExplorer [42], PIXEL [43] and.Psi4 [44] programs. Total energy (Etot) is decomposed into electrostatic (Eele), polarization (Epol), dispersive (Edis) and repulsion (Erep) components. The font's colors match those used for interaction energy values in Figures 7, 8, and 9 in the main text.

***$P2_1$*-but-OA**					
	E_{ele}	E_{pol}	E_{dis}	E_{rep}	E_{tot}
CE-B3LYP	-12.7	-5.1	-66.2	34.7	-53.4
PIXEL	-16.5	-8.8	-59.2	32.8	-51.6
SAPT0/jun-cc-pVDZ	-14.2	-4.9	-51.7	26.7	-44.1
SAPT0-d3mbj/jun-cc-pVDZ	-14.2	-4.9	-65.0	26.7	-57.5
ωB97M-V/def2-TZVPD	--	--	--	--	-52.9
	E_{ele}	E_{pol}	E_{dis}	E_{rep}	E_{tot}
CE-B3LYP	-7.0	-1.6	-63.0	31.5	-43.9
PIXEL	-7.7	-6.6	-56.9	33.9	-37.3
SAPT0/jun-cc-pVDZ	-6.7	-2.3	-45.0	22.2	-31.8
	E_{ele}	E_{pol}	E_{dis}	E_{rep}	E_{tot}
CE-B3LYP	-8.7	-2.5	-52.2	24.3	-41.6
PIXEL	-10.4	-5.4	-47.9	25.4	-38.2
SAPT0/jun-cc-pVDZ	-10.5	-2.6	-37.7	18.6	-32.2
	E_{ele}	E_{pol}	E_{dis}	E_{rep}	E_{tot}
CE-B3LYP	-3.9	-0.1	-28.4	15.3	-19.5
PIXEL	-4.6	-2.0	-26.2	14.9	-17.9
SAPT0/jun-cc-pVDZ	-3.4	-0.8	-19.8	10.6	-13.4
	E_{ele}	E_{pol}	E_{dis}	E_{rep}	E_{tot}
CE-B3LYP	-2.1	-0.3	-17..3	8.4	-12.2
PIXEL	-2.3	-1.4	-15.2	8.7	-10.2
SAPT0/jun-cc-pVDZ	-1.7	-0.5	-11.7	5.7	-8.2

*R*3-but-OA					
	Eele	Epol	Edis	Erep	Etot
CE-B3LYP	-11.6	-2.4	-71.3	38.8	-52.1
PIXEL	-13.2	-6.8	-65.7	40.1	-45.6
SAPT0/jun-cc-pVDZ	-10.6	-3.4	-52.5	26.9	-39.6
	Eele	Epol	Edis	Erep	Etot
CE-B3LYP	-9.3	-2.2	-66.6	34.6	-48.1
PIXEL	-10.1	-6.2	-60.7	36.0	-41.0
SAPT0/jun-cc-pVDZ	-9.1	-3.1	-49.4	24.9	-36.7
SAPT0-d3mbj/jun-cc-pVDZ	-9.1	-3.1	-65.4	24.9	-52.6
ωB97M-V/def2-TZVPD	--	--	--	--	-45.8
	Eele	Epol	Edis	Erep	Etot
CE-B3LYP	-7.9	-0.8	-55.0	23.8	-42.2
PIXEL	-9.6	-3.7	-49.6	25.1	-37.8
SAPT0/jun-cc-pVDZ	-7.6	-1.6	-37.9	16.6	-30.5
SAPT0-d3mbj/jun-cc-pVDZ	-7.6	-1.6	-53.9	16.6	-46.5
ωB97M-V/def2-TZVPD	--	--	--	--	-39.7
	Eele	Epol	Edis	Erep	Etot
CE-B3LYP	-1.9	-0.2	-17.9	10.3	-11.4
PIXEL	-2.0	-2.0	-16.2	11.0	-9.1
SAPT0/jun-cc-pVDZ	-0.9	-0.5	-11.9	6.2	-7.2
SAPT0-d3mbj/jun-cc-pVDZ	-0.9	-0.5	-17.3	6.2	-12.6
	Eele	Epol	Edis	Erep	Etot
CE-B3LYP	-0.3	-0.1	-9.4	3.0	-6.6
PIXEL	-0.1	-0.4	-8.0	2.8	-5.8
SAPT0/jun-cc-pVDZ	0.1	-0.2	-6.3	2.2	-4.3
SAPT0-d3mbj/jun-cc-pVDZ	0.1	-0.2	-9.0	2.2	-6.9
	Eele	Epol	Edis	Erep	Etot
CE-B3LYP	-24.8	-5.4	-22.2	30.3	-30.8
PIXEL	-29.5	-10.0	-21.8	29.1	-32.2
SAPT0/jun-cc-pVDZ	-27.4	-6.4	-17.2	27.4	-23.6
SAPT0-d3mbj/jun-cc-pVDZ	-27.4	-6.4	-23.1	27.4	-29.5
ωB97M-V/def2-TZVPD	--	--	--	--	-25.8
	Eele	Epol	Edis	Erep	Etot
CE-B3LYP	-26.0	-6.5	-19.1	32.0	-29.2
PIXEL	-31.9	-11.8	-19.5	31.0	-32.2
SAPT0/jun-cc-pVDZ	-21.8	-4.6	-17.2	22.8	-20.8
SAPT0-d3mbj/jun-cc-pVDZ	-21.8	-4.6	-24.3	22.8	-27.9
ωB97M-V/def2-TZVPD	--	--	--	--	-24.4
	Eele	Epol	Edis	Erep	Etot
CE-B3LYP	-7.9	-0.4	-55.0	37.4	-33.5
PIXEL	-8.7	-6.2	-50.2	38.6	-26.6
SAPT0/jun-cc-pVDZ	-5.3	-1.9	-38.9	23.8	-22.4
	Eele	Epol	Edis	Erep	Etot
CE-B3LYP	-5.1	-1.6	-39.1	16.6	-30.4
PIXEL	-5.9	-3.5	-33.7	15.8	-27.2
SAPT0/jun-cc-pVDZ	-6.2	2.1	-30.2	-13.0	-25.5
	Eele	Epol	Edis	Erep	Etot
CE-B3LYP	-4.0	-1.5	-32.2	12.6	-25.6
PIXEL	-4.4	-2.9	-27.8	12.0	-23.2

SAPT0/jun-cc-pVDZ	-4.4	-1.7	-24.6	9.3	-21.5
	**E_{ele}**	**E_{pol}**	**E_{dis}**	**E_{rep}**	**E_{tot}**
CE-B3LYP	-9.4	-0.6	-57.8	41.9	-34.8
PIXEL	-10.2	-7.3	-54.8	43.8	-28.5
SAPT0/jun-cc-pVDZ	-6.5	-2.2	-41.2	26.9	-22.9

Table S5. Percentage contribution of H···H, O···H and C···H contacts to the Hirshfeld surface area.

Note: Calculated based on: * Langer [34]; ** Beseda [35].

	H···H	O···H	C···H
***R*3-but-OA**			
Molecule A	91.9	8.0	0.1
Molecule B	92.2	7.6	0.1
P*2$_1$–but-OA	92.2	7.5	0.3
**Me-GE****	85.0	14.2	0.7
**Me-11-oxo-OA**			
Molecule A	85.5	13.5	0.8
Molecule B	84.8	14.2	0.9
